# A multi-criteria approach to investigate spatial distribution, sources, and the potential toxicological effect of polycyclic aromatic hydrocarbons (PAHs) in sediments of urban retention tanks

**DOI:** 10.1007/s11356-022-24168-1

**Published:** 2022-11-17

**Authors:** Nicole Nawrot, Anna Pouch, Karolina Matej-Łukowicz, Ksenia Pazdro, Muhammad Mohsin, Shahabaldin Rezania, Ewa Wojciechowska

**Affiliations:** 1 Gdansk University of Technology, Faculty of Civil and Environmental Engineering, Narutowicza 11/12, 80-233 Gdansk, Poland; 2 Institute of Oceanology of the Polish Academy of Sciences, Marine Geotoxicology Laboratory, Powstańców Warszawy 55, 81-712 Sopot, Poland; 3 School of Forest Sciences, University of Eastern Finland, Yliopistokatu 7, P.O. Box 111, 80100 Joensuu, Finland; 4 Department of Environment and Energy, Sejong University, Seoul, 05006 South Korea

**Keywords:** Sediments, Polycyclic aromatic hydrocarbons, Retention tanks, Urban catchment, Environmental contamination, Contamination sources

## Abstract

Bottom sediments deposited in retention tanks (RTs) located on two urban streams (Oliwski and Strzyza) in the central part of Gdansk (Poland) were analysed for polycyclic aromatic hydrocarbons’ (PAHs) content. PAHs were extracted from samples with methylene chloride, then the extracts were subjected to clean-up applying the solid phase extraction (SPE) method. Quantitative and qualitative determination of 16 PAHs was performed with the use of gas chromatography/mass spectrometry technique. A multi-dimensional approach was applied to analyse PAHs’ spatial distribution, source, and contamination status. Potential sources of PAHs were verified using isomer ratios supported by a statistical approach. The Σ16PAHs (in mg/kg d.w.) ranged from 1.95 ± 0.64 to 20.4 ± 6.8 for RTs located on the Oliwski Stream and from 0.50 ± 0.17 to 8.6 ± 2.9 for RTs located on the Strzyza Stream. PAHs detected in bottom sediments were mainly composed of 4- and 5-ring compounds. PAH isomer ratios such as Phen/Anth, Flth/Pyr, B(a)A/B(a)A + Chry, Inpy/Inpy + B(ghi)P, Flth/Flth + Pyr, Anth/Anth + Phen, and Flth/Flth + Pyr suggested delivery pathways for biomass, coal, and petroleum combustion. Petrogenic PAHs related to fuel leaks from cars were not detected. Statistical analyses confirmed traffic and heating system sources, while factor analysis (FA) pointed out the abrasion of wasting parts of vehicles. Based on threshold levels presented in sediment quality guidelines (SQGs), in most cases, PAHs were at low levels with occasional negative biological effects on organisms. Only sediments deposited in two RTs located on the Oliwski Stream presented harmful features for sediment-dwelling organisms. The risk assessment performed accordingly to the Σ16PAHs presented a moderate and high risk for biota. This study not only reflects the direct threat related to PAH content in bottom sediments, but also highlights the overall pollution of an area, considered to be a recreational part of the city (Oliwski Stream catchment). The findings of this study highlight the need to launch preventative methods to protect the area against pollution from heating system emission and traffic.

**Supplementary Information:**

The online version contains supplementary material available at 10.1007/s11356-022-24168-1.

## Introduction

Anthropogenic activities in urban and industrial areas are commonly recognised as contributors of different types of pollutants. Transportation and energy production (including central and local heating systems) are undoubtedly main culprits in terms of polycyclic aromatic hydrocarbons (PAHs), which are as acknowledged to be substances of great environmental concern. PAHs are ubiquitous in the environment, susceptible to bioaccumulation, and above all exhibit teratogenic and carcinogenic properties (Meng et al. 2019). These PAHs attributes have landed them on the US Environmental Protection Agency’s (US EPA) list of priority control pollutants (Melnyk et al. 2015).

Natural pathways of origin include volcanic activity, forest, grass and bush fires, natural spills of petroleum, erosion of bituminous rocks, as well as transformation of organic material in recent sediments and soils (Lubecki and Kowalewska 2010). Anthropogenic sources of PAHs include incomplete combustion of fossil fuels (i.e. coal and petroleum) and other organic materials such as wood (incomplete burning) as well as abrasion of coal-tar-based pavement sealant and asphalt (Witter et al. 2014). PAHs are present in different components of the environment: air, soil, or water bodies (Kim et al. 2013), seawater (Pouch et al. 2021), and bottom sediments (Pouch et al. 2017), and they spread throughout all geographical regions (Dudhagara et al. 2016; Gdara et al. 2017; Li et al. 2018; Montuori et al. 2016; Szopińska et al. 2019). Atmospheric deposition, wastewater, fuel and oil leakage, and surface runoff from impervious surfaces have all been identified as PAH routes to aquatic environments (Witter et al. 2014). PAHs are easily adsorbed on solids and colloids when transferred into the water column (Crane 2014), similarly to other pollutants like trace metals (TMs). The major mechanism of PAHs deposition is sorption on suspended matter either organic or mineral, followed by suspended matter sinking and deposition to bottom sediments (that is influenced by compound hydrophobicity assessed by octanol/water partition coefficient increasing with growing molecular weight) (Revitt et al. 2014; Wojciechowska 2013). Furthermore, PAHs (mostly low molecular weight-less hydrophobic) can become re-suspended in water as a consequence of physical, chemical, and biological processes occurring at the sediment/water interface. Therefore, bottom sediments may be considered from two perspectives: as a retention material (collector/settler) and as a secondary source of pollutants from different classes (Bąk et al. 2019). Dredged material (bottom sediments), that is routinely removed from urban retention tanks (RTs) to keep their proper retention capacity, may be reused in the environment (Matej-Łukowicz et al. 2021). This is of particular concern because it may pose a threat to people and living organisms.

Environmental spreading and threat caused by PAHs is recognised internationally and forces international institutions to set requirements to reduce PAH pollution. The European Commission has specified a reduction in emissions in Europe by 50–55% by 2030 (compared to 1990) and to reach full carbon neutrality by 2050 (European Commission 2019) by implementing, for instance, Regulation EU 1189/2015 which sets standards for the eco-design of solid fuel boilers. Poland is the largest hard coal and second-largest lignite producer in the EU, generating around 80% of its electricity from coal (Brauers and Oei 2020), which definitely highlights the need to monitor PAHs spreading in the environment. Among the PAHs emitted in various parts of the environment, about 89.9% is accumulated in the soil, while 9.3% is accumulated in bottom sediments, 0.5% is present in the air, and 0.3% in surface waters (Wang et al. 2018). Bottom sediments serve as sinks and carriers for contaminants in aquatic environments, and thus reflect the area's overall contamination status (Jaskuła and Sojka 2022).

Relatively scarce data exist in terms of PAH' occurrence and distribution pathways in bottom sediments of urban RTs that collect surface runoff and work as receivers of the stormwater drainage system (SDS). The sediments deposited in urban water bodies collecting stormwater runoff pose a fingerprint of the contaminants delivered from the urban area, which largely indicates the level of contamination of the water, soil, and atmosphere in the region in question. Co-occurrence and co-existence of PAHs and TMs are very common in urban watersheds, especially in the receivers of industrial and domestic wastewater discharges as well as stormwater discharges containing road deposited sediments, which is at least partly due to the fact that they often originate from the common anthropogenic source (fuel or coal burning or traffic) (Eeshwarasinghe et al. 2019; Nawrot et al. 2020a). These compounds bound in bottom sediments create a major hazard to living organisms and can cause changes in local biota (Bąk et al. 2019).

The main objectives of this study are to 1) assess the 16 EPA-listed PAHs spatial distribution, and contamination in bottom sediments collected from receivers of the SDS in urban RTs; 2) detect the potential sources of PAHs focused on urban associated activities and verification of the origin of PAH in the context of PAH ratios and correlations with TMs bound in bottom sediments; and 3) evaluate the potential toxicological and biological impacts on humans and the aquatic environment. This is the first study discussing PAHs contamination of the bottom sediments in urban RTs in Poland, as well as the first case study for Gdansk area. The levels of PAHs detected in the RTs were compared with those of the nearby lakes and with PAHs pollution in the coastal zone of the Gulf of Gdansk—the final receiver of the Oliwski and Strzyza. The data presented in the manuscript not only reflects the direct threat associated with PAHs content in bottom sediments, but it also contributes to an assessment of the overall pollution scenario of the area, which may be useful as a benchmark for future research and serve environmental management by limiting the spread of potentially toxic compounds. PAHs concentrations in bottom sediments of water bodies mostly reflect the status of atmospheric pollution, which is one of hot environmental and social issues in Poland. The measurements conducted on sediments may be less sensitive to temporal fluctuations caused for instance by weather conditions than measurements of air quality. Recent efforts to mitigate air pollution by replacing old-type coal-fired heating systems and promoting renewable energy sources may be terminated due to emerging energy crisis. Therefore, the results of this study can provide a benchmark for assessment of air pollution mitigation strategies.

## Material and methods

### Retention tank selection and site description

**Fig. 1:**
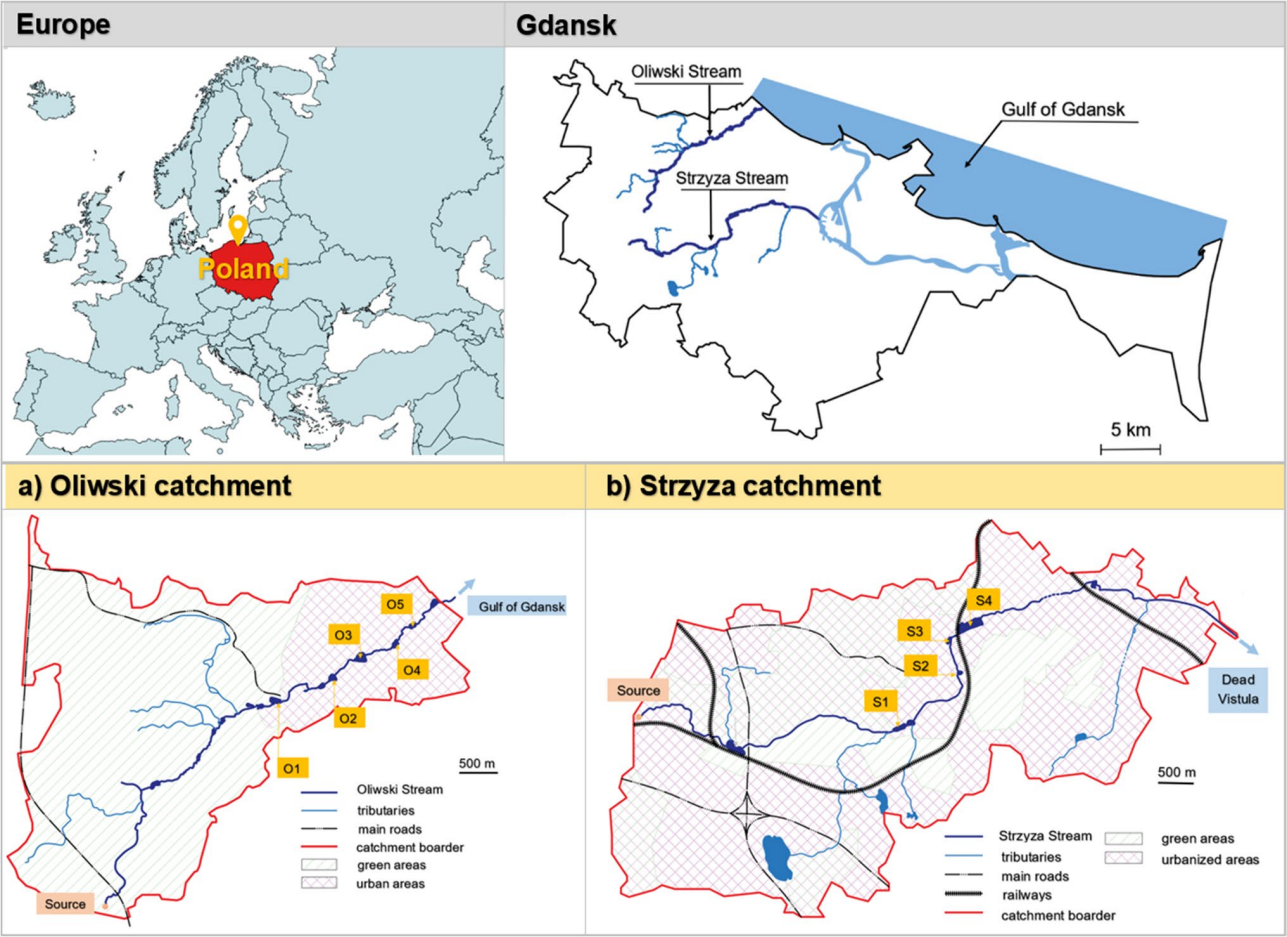
Location of the analysed retention tanks (RTs) and sampling points on the map of a) Oliwski catchment (O1—Spacerowa RT, O2—Grunwaldzka RT, O3—Subisława RT, O4—Chłopska RT, and O5—Orlowska RT) and b) Strzyza catchment (S1—Nowiec RT, S2—Ogrodowa RT, S3—Potokowa RT, and S4—Srebrniki RT)

Sediment samples were collected between June and August 2017 from RTs located on two urban streams in Gdansk (Strzyza and Oliwski) presented in Fig. 1. In Gdansk, due to the large attitude difference, there is a high risk of flooding induced by torrential rainfalls. To address this risk, a series of retention ponds or RTs are constructed on urban streams that also function as recreational areas. At the same time, both urban streams and RTs act as receivers of the stormwater network. As a result, a complex stormwater system based partly on underground sewers and partly on streams and open RTs is formed.

In this study, nine RTs located on two main urban streams that flow through the central part of the city (Oliwski and Strzyza) were analysed. The characteristics of the streams and the development around the RTs were described by Nawrot et al. (2020b) and Wojciechowska et al. (2019). The sampling points locations were selected on the basis of the field study of Gdansk to identify “hot points”. We choose two streams that flow through the most developed parts of the city, where a lot of urban activities associated with potential PAHs pollution hot spots exist. Generally, sampling points were located at the vicinity of busy traffic arteries and/or in the older districts, where heating system is predominantly based on older-type individual solid fuel boilers. Earlier studies (Nawrot et al. 2020b; Wojciechowska et al. 2019) focused on TM pollution status and source apportionment in the area of question, showing that both traffic and coal-fired individual boilers are contributing to TM enrichment of sediments deposited in RTs of two investigated watercourses. Figure 1 presents the location of five RTs located in the middle run of the Oliwski Stream (O1—Spacerowa RT, O2—Grunwaldzka RT, O3—Subisława RT, O4—Chłopska RT, and O5—Orlowska RT) as well as four RTs located in the middle run of the Strzyza Stream (S1—Nowiec RT, S2—Ogrodowa RT, S3—Potokowa RT, and S4—Srebrniki RT). S1 RT is located directly beneath Landscape Park and is only fed with stream waters passing the forested area, without any relevant anthropogenic activities. Because there is no direct stormwater discharge to this RT, a negligible sediment contamination was expected. In total, 12 sampling points were located in the analysed RTs (two sampling points at O2, S2, and S3; one sampling point at other RTs). Location, area, retention capacity, normal and maximum damming level, grain size (sand, silt, and clay), and organic matter (OM) content in sediment samples are shown in Tab.S1.

### Sampling and measurements

Surface sediment samples (0–5 cm) were collected at sampling points in each RT presented in Fig. 1 (O1–O5 and S1–S4). Sample collection was carried out at a 5-m distance from the inflow (IN) to the RT (measured in a straight line). Additionally, sediment samples close to the outflow (OUT) were collected in the O2, S2, and S3 RTs. A grab sampler was used to collect sediment samples. One sample consisted of sub-samples collected from 3 crosswise located sites (within the 0.25 m^2^ area of the sampling point). The samples were placed in clean polyethylene packages and delivered to the laboratory within 4 h of sampling. The samples were freeze-dried, and after removing the stones, they were homogenised using a mortar and pestle.

The determination of PAHs was performed according to the procedure described by Bogdaniuk et al. (2012). Briefly, the sediments were soaked for 24 h with acetone. The contaminants were extracted with methylene chloride in a shaker for 24 h. The extracts were evaporated with a N_2_ stream to 1 cm^3^. Next, clean-up was performed by absorption chromatography on SPM columns (SiO_2_) and activated copper. Methylene chloride was used to extract PAHs. The extracts were concentrated under a gentle flow of nitrogen to 1 cm^3^. Gas chromatography-mass spectrometry (GC–MS) with HP 6890 gas chromatograph equipped with a 60 m × 0.32 mm I.D. fused silica capillary column, coated with bonded a 0.5 μm nonpolar DB-5 phase (JW), and model MSD HP 5973 detector operated in the selected ion monitoring (SIM) mode was used to analyse PAHs. All analyses were performed in three replicates, and the results for the analysed sediments were given on a dry weight basis. Blank samples were measured according to the same procedure.

Granulometric analysis was performed with the use of six normalising sieve apertures (< 0.0625 mm, 0.125 mm, 0.250 mm, 0.500 mm, 1.0 mm, and 2.0 mm). 250 g of dried sediments were sieved in 15 min in a mechanical sieve shaker. The residuals on each of the sieves were weighed (0.01 g accuracy).

The total organic matter (OM) content in the sediments was determined by combustion of dried sediments at 450 °C for 2 h in the muffle furnace (carbolite). It was assumed that the loss incurred by heating corresponds to the share of organic substances in the analysed samples.

### Quality assurance/quality control (QA/QC)

The standard mixture contained all 16 PAHs, as recommended by the US EPA, in dichloromethane (2,000 μg/cm^3^ for each compound; Restek Corporation, USA). The PAH standards consisted of 16 USEPA priority PAHs: Napt, Acny, Acen, Flur, Flth, Anth, Phen, Pyr, Chry, B(a)A, B(b)F, B(a)P, B(k)F, Inpy, D(ah)A, and B(ghi)P. PAH concentrations were calculated using external calibration curves plotted for each compound in the linear range of the detector response. The detection and qualification limits of this method were 0.0003 and 0.001 mg/kg dry weight (d.w.) respectively. The quantification method and the entire analytical procedure were applied several times to reference material LGC6188 (River Sediment—PAHs). Recoveries of individual compounds ranged from 60 to 120% depending on the compound. The related data of QA/QC are provided in Tab.S.2.

### Ecotoxicological effect on biota

Indicators commonly used in sediment quality guidelines (SQGs) were applied to assess toxic concentrations of contaminants in the sediments studied. In particular, the measured concentrations were compared to threshold effect concentrations (TEC) and probable effect concentration (PEC) values established by MacDonald et al. (2000). PAHs’ concentrations below TEC, between TEC and PEC, and above PEC indicate that biological effects are not expected, are harmful occasionally, and are harmful frequently, respectively.

To verify the risk of the impact of individual PAHs on aquatic biota, the risk quotient (RQ) coefficient (Eq. 1) can also be used. In this study, RQ values were calculated applying the minimum and maximum permissible PAH concentrations in sediments (negligible concentrations—NCs and maximum permissible concentrations—MPCs, respectively) (Kalf et al. 1997) (Eqs. 2 and 3 respectively).

1 $$RQ=\frac{{C}_{PAH}}{{C}_{QV}}$$

2 $${RQ}_{NCs}=\frac{{C}_{PAH}}{{C}_{QV(NCs)}}$$

3 $${RQ}_{MPCs}=\frac{{C}_{PAH}}{{C}_{QV(MPCs)}}$$

where ${C}_{PAH}$ is the concentration of individual PAH in the sediment and ${C}_{QV}$ is the corresponding quality values of individual PAH. ${C}_{QV(NCs)}$ is the quality value for the PAH NCs in the sediment and ${C}_{QV(MPCs)}$ is the quality value for the PAH MPCs in the sediment. ${C}_{QV(NCs)}$ for individual PAHs in sediment samples proposed by Cao et al. (2010) are as follows (in ng/g d.w.): Napt—1.4, Acny—1.2, Acen—1.2, Flur—1.2, Phen—5.1, Anth—1.2, Flth—26, Pyr—1.2, B(a)A—3.6, Chry—107, B(b)F—3.6, B(k)F—24, B(a)P—27, Inpy—59, D(ah)A—27, and B(ghi)P—75. ${C}_{QV(MPCs)}$ is assessed to be 1000 times greater than ${C}_{QV(NCs)}$. According to Cao et al. (2010), ${RQ}_{NCs}$ < 1 indicates negligible concern of individual PAH, while ${RQ}_{NCs}$ ≥ 1 implies moderate risk. At the same time ${RQ}_{MPCs}$ < 1 means moderate risk, while ${RQ}_{MPCs}$ ≥ 1 indicates high risk. With ${RQ}_{NCs}$ ≥ 1 and ${RQ}_{MPCs}$ < 1 there is a need for control measures or remedial actions to be undertaken (Gdara et al. 2017). Based on ${RQ}_{NCs}$ and ${RQ}_{MPCs}$ for individual PAHs which were not less than 1, the risk for total PAHs was calculated as ${RQ}_{\Sigma PAHs(NCs)}$ and ${RQ}_{\Sigma PAHs(MPCs)}$, respectively, according to the method proposed by Cao et al. (2010).

### PAH sources’ tracking

Potential sources of PAHs detected in the collected samples were assessed using isomer ratios. Various thermodynamic properties of PAH isomers can be used to assess sources of PAHs in environmental samples. The diagnostic ratios can change during phase transfers and depending on environmental factors. Therefore, to get reliable data, the common method is to compare different indicators. Phen is more thermodynamically stable compared to Anth and so a high Phen/Anth (> 10) ratio indicates petrogenic origin. Among PAHs with 4 rings in the molecule, B(a)A is more thermodynamically stable than Chry. A B(a)A/(B(a)A + Chry) ratio between 0.2 and 0.35 indicate a coal combustion origin, ratio < 0.2 means petrogenic origin, while ratio > 0.35 means pyrogenic related sources (Tobiszewski and Namieśnik 2012). Yunker and Macdonald (2003) defined the Inpy/(Inpy + B(ghi)P) ratio which indicates a petrogenic source when < 0.2, a petroleum combustion source when the ratio ranges 0.2–0.5, and a grass, wood, and coal combustion source when > 0.5. According to De La Torre-Roche et al. (2009) the Flth/(Flth + Pyr) ratios indicate a petrogenic source when the ratio is < 0.4, fossil fuel combustion for ratios between 0.4–0.5, and grass, wood, coal combustion for > 0.5. Anth/Anth + Phen greater than 0.1 means a combustion-related source, while less than 0.1 means a petroleum source (De La Torre-Roche et al. 2009).

### Statistical analyses

Principal component analysis (PCA) was used to reduce the number of variables and verify the relationship between PAHs (as well as distinguish PAH sources). PCA may be useful for the semiquantitative description of the contributions of main sources (Gdara et al. 2017). First, the eigenvalues and loads were calculated, and the scree plots were made, which allowed us to determine the number of new variables. Subsequently, the factor coordinates of the cases and the factor coordinates of the variables (loads) were determined. PCA is a transformation method of observed variables into new, orthogonal variables, the so-called principal components (PCs). The main aim of PCA is to represent the total variation of the PAHs with the minimum possible number of factor loadings. PCA was performed with Statistica 13 software (Statsoft, Poland). Factor analysis (FA) was used to understand relationships between PAHs and TMs in bottom sediments deposited in RTs of two analysed catchments. The correlations between PAHs and TMs were implemented to verify similarities which may indicate potential common sources. The FA procedure is similar to PCA and includes the selection of variables, the determination of the correlation matrix (covariance), the validation of the factor analysis, the choice of the factor model, the determination of the main factor (based on the scree plot), rotation, and the interpretation. The interpretation of PCA and FA relies on analysing loadings. Loads close to − 1 or 1 indicate that the PC or factor strongly influences the variable. Loads close to 0 indicate that the PC or factor has a weak influence on the variable. Some variables may have high loadings on multiple PCs or factors.

### Toxicity assessment to humans

The toxic equivalent quotient (TEQ) based on B(a)P equivalent concentration is a widely used method to assess the harmfulness of PAHs to human health (Nisbet and LaGoy, 1992). B(a)P is assumed to be the reference compound for assessment of the carcinogenicity potency of other PAHs. The toxicity of the each of 16 measured PAHs was evaluated by its toxicity equivalency factor (TEF), which is related to the toxicity, mutagenicity, and carcinogenicity of subsequent PAHs. The TEQ value is the sum of the products of the concentrations of the individual PAHs and their relative TEF (Eq. 4).

4 $$TEQ=\sum_{i=1}^{n}{C}_{i}\bullet {TEF}_{i}$$

where ${C}_{n}$ is the concentration of PAHs, while the TEF for PAHs is as follows: Acen = 0.001, Acny = 0.001, Anth = 0.01, B(a)A = 0.1, B(a)P = 1, B(b)F = 0.1, B(ghi)P = 0.01, B(k)F = 0.1, Chr = 0.01, D(ah)A = 1, Flth = 0.001, Flur = 0.001, Inpy = 0.1, Phen = 0.001, Pyr = 0.001, Napt = 0.001 (Amjadian et al. 2016). A TEF value of 0 indicates that the compound does not have carcinogenic activity, while a TEF of 1 indicates high carcinogenicity.

## Results and discussion

### PAHs’ distribution

The average concentrations of Σ16PAHs in the bottom sediment samples collected from RTs on the Oliwski and Strzyza streams are shown in Table 1, while the SD values are presented in Tab.S.3. The sum of Σ16PAHs (in mg/kg d.w.) in bottom sediments samples changed in the range from 1.95 ± 0.64 to 20.4 ± 6.8 for the RTs located on the Oliwski Stream and from 0.50 ± 0.17 to 8.6 ± 2.9 for the RTs located on the Strzyza Stream. In general, 15 target PAHs were detected in all samples (the exception was Napt, with concentration below the detection limit in sample S2). Considering the median concentration of 3.4 mg/kg d.w., the highest content of Σ16PAHs was observed in sediments collected at sampling points O1, O2 (IN and OUT) and S3 (IN and OUT). The sum of 7 probable human carcinogenic PAHs (Σ7CPAHs, including B(a)A, Chry, B(b)F, B(k)F, B(a)P, Inpy, and D(ah)A) varied between 0.18 and 8.87 mg/kg d.w. (median 1.57 mg/kg d.w.) and made up to 36–61% of the total Σ16PAHs (mean = 44%). The presence of contaminants in sediments, in addition to their sources, depends on the structure of the sediment (e.g. organic matter content, porosity, or grain size) (Mechelińska et al. 2009). It has been widely reported that PAHs have an affinity for OM content, resulting in a relationship whereby the higher the OM content, the higher the PAH content. Generally, sediments in the Oliwski Stream were characterised with the highest OM content in O1, O2 IN, and O2 OUT, which is consistent with the highest Σ16PAHs (20.39, 16.87 and 5.11 mg/kg d.w., respectively). Despite this, the lack of statistically significant correlation between PAHs concentrations and OM (*p* > 0.05) as well as PAHs concentrations and fine fraction content (*p* > 0.05) indicates that the distribution of PAHs in bottom sediments in the studied RTs is not relevantly influenced by those properties. The lack of correlation between PAHs concentrations and fine fraction content could be explained by relatively low grain size variation in the collected samples. All collected sediments were classified as sand or muddy sand. Moreover, physico-chemical properties of the PAHs group (represented by, e.g. log Kow values) are much more diverse than, for example in the case of PCBs, what may influence binding to OM in sediment (Nam et al., 2008). This may partly explain lack of statistically significant dependencies between OM and pollutant contents. On the other hand, low correlations between PAHs and sediment granulation may not only be due to the adsorption capacities of individual PAHs but may also result from processes occurring in the water column and at the interface water/sediment during deposition/resuspension including mixing, partitioning, and microbial degradation.

**Table 1:** The average concentration of PAHs [mg/kg d.w.] in urban streams sediments (Oliwski and Strzyza) in Gdansk, Poland, and the data of sediment quality guidelines (SQG) values according to MacDonald et al. (2000)

PAH [mg/kg d.w.]	Oliwski Stream						Strzyza Stream						Min	Max	Median	SQG values	
	O1	O2	O2	O3	O4	O5	S1	S2	S2	S3	S3	S4					
																TEC*	PEC*
	IN	IN	OUT	IN	IN	IN	IN	IN	OUT	IN	OUT	IN					
Napt	0.092	0.15	0.039	0.02	0.004	0.049	0.001	< LD	0.013	0.063	0.048	0.012	< LD	0.15	0.044	0.176	0.561
Acny	0.06	0.076	0.017	0.023	0.004	0.017	0.002	0.006	0.021	0.04	0.032	0.009	0.002	0.076	0.019	-	-
Acen	0.229	0.057	0.048	0.024	0.009	0.021	0.005	0.017	0.011	0.03	0.024	0.03	0.005	0.229	0.024	-	-
Flur	0.248	0.125	0.055	0.037	0.014	0.038	0.009	0.019	0.018	0.072	0.059	0.036	0.009	0.248	0.038	0.0774	0.536
Phen	**2.76**	**1.3**	0.59	0.40	0.143	0.323	0.082	0.187	0.271	0.48	0.45	0.269	0.082	**2.76**	0.362	0.204	1.170
Anth	0.54	0.32	0.115	0.070	0.042	0.058	0.01	0.035	0.047	0.117	0.099	0.049	0.01	0.54	0.064	0.0572	0.845
Flth	**3.6**	**2.81**	0.96	0.710	0.277	0.58	0.112	0.296	0.6	**1.41**	**1.18**	0.38	0.112	**3.6**	0.655	0.423	2.230
Pyr	**2.95**	**2.09**	0.76	0.570	0.227	0.48	0.088	0.245	0.44	**1.22**	**1.00**	0.301	0.088	**2.95**	0.525	0.195	1.520
B(a)A	**1.53**	**1.19**	0.39	0.280	0.119	0.235	0.038	0.125	0.29	0.54	0.45	0.148	0.038	**1.53**	0.285	0.108	1.050
Chry	**1.73**	**1.57**	0.49	0.360	0.144	0.34	0.054	0.165	0.41	0.82	0.72	0.184	0.054	**1.73**	0.385	0.166	1.290
B(b)F	**1.34**	**1.44**	0.36	0.224	0.076	0.251	0.023	0.09	0.34	0.75	0.62	0.106	0.023	**1.44**	0.296	-	-
B(k)F	**1.42**	**1.28**	0.34	0.279	0.092	0.266	0.03	0.112	0.36	0.66	0.54	0.122	0.03	**1.42**	0.310	-	-
B(a)P	**1.37**	**1.27**	0.317	0.229	0.72	0.207	0.022	0.09	0.302	0.57	0.48	0.106	0.022	**1.37**	0.310	0.150	1.450
Inpy	**1.22**	**1.58**	0.295	0.171	0.034	0.229	0.011	0.062	0.35	0.82	0.66	0.067	0.011	**1.58**	0.262	-	-
D(ah)A	0.257	0.296	0.049	0.029	0.007	0.035	0.002	0.011	0.066	0.168	0.13	0.011	0.002	0.296	0.042	0.033	-
B(ghi)P	**1.04**	**1.32**	0.284	0.192	0.04	0.248	0.016	0.054	0.297	0.84	0.64	0.079	0.016	**1.32**	0.266	-	-
∑PAHs	**20.39**	**16.87**	**5.11**	**3.62**	**1.95**	**3.38**	0.505	**1.51**	**3.84**	**8.60**	**7.13**	**1.91**	0.5	20.4	3.40	1.610	22.800

< LD below the limit detection.

**Bold**—values with a concentration above 1 mg / kg d.w

*TEC threshold effect concentration, *PEC* probable effect concentration (MacDonald et al. 2000).

In the Strzyza Stream, the highest concentrations were recorded for Flth and Pyr in sediments deposited in S3 RT. High concentration of PAHs is frequently found in the bottom sediments of urban regions due to the location of most PAHs sources in and/or near urban centres (Yuan et al. 2021). In the case of RTs analysed on the Strzyza Stream, an increasing impact of anthropopressure and urbanization of the area from S1 to S4 can be distinguished. The S2, S3 and S4 RTs are located close to a main road and transportation junctions in Gdansk and thus receive a large part of the surface runoff from these streets. In addition, two stormwater network interceptors’ inflow directly to S3. In the vicinity of S2–S4, a railway line is located, operated by Diesel Multiple Units (Szmagliński et al. 2021), which could be another potential source of PAHs. According to Levengood et al. (2015), who analysed bottom sediments collected from waterways associated with a suburban railway in Chicago, PAHs’ concentrations were on the convergent level to our study (eg. max concentrations in mg/kg d.w. for Phen—0.56, Pyr—0.58, B(a)A—0.32, B(a)P—0.33, Chry—0.63, and Flth—0.62). The presence of major roads and a railway line in the vicinity of the study area may supplement PAHs originating from different means of transportation.

In the case of the Oliwski Stream, there is no obvious trend regarding the spatial distribution of PAHs in subsequent RTs. In this catchment, all analysed RTs are located in an urban area within a close distance to potential sources of PAHs. However, O1 and O2 are located directly next to main roads with intensive traffic. The spatial development of these catchment areas, with particular focus on the presence of paved surfaces in the vicinity of both streams, was investigated by Wojciechowska et al. (2019) in the context of contamination of bottom sediments with TM. The results clearly showed the correlation between impervious areas in the catchment area and the increased contamination of bottom sediments with TMs.

Nielsen et al. (2015) analysed stormwater samples originating from highway runoff. They indicated that a substantial amount of PAHs is found in filtrated fractions (0.7 µm filtration), which are difficult to remove by conventional stormwater techniques such as sedimentation ponds and a chain of sedimentation chambers. This study also pointed out the importance of developing advanced techniques for stormwater treatment to reduce the dissolved PAHs and, subsequently, decrease secondary water pollution due to the leaching of PAHs from the deposited sediments. Research carried out in south-central Pennsylvania, USA (Conodoguinet Creek, a small urbanising watershed), demonstrates that coal-tar-based seal coat is a major PAH source in urban freshwater stream sediments (Witter et al. 2014). The maximum concentrations of PAHs obtained in this study are similar to data reported by Weinstein et al. (2010) in coastal stormwater ponds sediments at the residential low-density area (e.g. max reported concentration in mg/kg d.w. for Chry—2.149, B(a)A—1.288, Phen—1.511, and Flth—6.433), while definitely lower than observed by the same authors in a commercial area (just to mention a few of the max reported concentration in mg/kg d.w. for Chry—7.328, B(a)A—6.867, Phen—14.789, and Flth—36.123).

In addition, we compared PAHs’ concentrations in RTs in the Oliwski and Strzyza streams with data available for sediments in the lakes of the nearby district and for sediments in the coastal zone of the Gulf of Gdansk (Baltic Sea)—the final receiver of both streams. The concentrations of PAHs in freshwater and marine sediments represented a quite wide range of values (Tab.S4). The PAHs’ concentrations measured in our study were generally higher than detected in wooded areas with less anthropogenic impact on the environment (e.g. Radunskie Lake, Tab.S4). Only the lower range of Σ16PAHs’ concentrations detected in our study in RTs located in less urbanized parts of the city were at a level comparable to some of the higher concentrations of Σ12PAHs measured in Radunskie Lake (Tab.S4, Czarnecka 2012). On the other hand, the highest concentrations of Σ16PAHs in our study were higher than those detected in heavily polluted surface sediments collected in 2011 from the Klasztorne Male Lake in the city of Kartuzy (Tab.S4, Tylman et al. 2013). The concentration of Σ16PAHs in sediments from Druzno Lake (1.528 mg/kg d.w.) located near Elblag (a city around 80 km east from Gdansk with more than a hundred thousand inhabitants) and the Warsaw-Gdansk Expressway was comparable to the lower concentrations detected in sediments from the studied RTs. Bojakowska et al. (2012) reported a median of 3.15 mg/kg d.w calculated for sediment samples collected from 150 Polish lakes in 2009, with Flth and Pyr being dominant compounds, which is quite similar to our findings. When comparing our results with those reported by the Polish Chief Inspector of Environmental Protection (2017), the concentrations of Σ17PAHs detected in sediments in 2018–2019 from Polish rivers and lakes, except the Ostrowickie and Czluchowskie lakes, were lower than those detected in our investigation (Tab.S4). According to Bojakowska et al. 2012 and CIEP (2019), high levels of PAH were found mainly in lakes located in cities or on their outskirts. This leads to a conclusion that bottom sediment samples investigated in this study exhibited higher PAHs concentrations against sediments from water bodies in Poland.

Due to low PAHs solubility, the major process of PAHs removal is adsorption to solids followed by deposition to the sediments (Wojciechowska 2013). Release of buried pollutants into the water can take place when resuspension of bottom sediments occurs, like during flood episodes or sediment dredging works. Strzyza and Oliwski are relatively short streams with several RTs located one after another to prevent flooding of urbanized districts. The contaminants brought with surface runoff, inflow of storm sewers and atmospheric deposition to streams’ water are deposited in bottom sediments. However, on flooding occasions, transport of TM from RTs located in the upper stream parts to those located in the middle or lower part was observed in the past on Oliwski Stream (Wojciechowska et al. 2017). The same scenario can be expected in the case of PAHs. Usanase et al. (2021) performed sediment distribution monitoring during the settling process and identified that sediment particles < 20 µm remain in the water column up to 20 min after resuspension, posing a threat of PAH mobilisation. This event-based migration of deposited contaminants to the lower parts of the streams is likely to end-up in reaching the sea and year-by-year growth of the contaminants concentrations that would pose risk to the Baltic Sea ecosystem. The concentration of Σ12PAHs in the Gulf of Gdansk ranged from much lower levels than detected in our study (0.009 mg/kg d.w., Lubecki and Kowalewska 2010) to quite high (170.00 mg/kg d.w.) near a former location of municipal WWTP outlet (Falandysz et al. 2006) (Tab.S4). Lubecki and Kowalewska (2010) reported mainly less polluted sediment than RT sediment samples. In general, this comparison confirms that RTs located on streams in the coastal area can be considered as a potential source of pollution for the Baltic Sea, especially during extreme weather phenomena episodes.

**Fig. 2:**
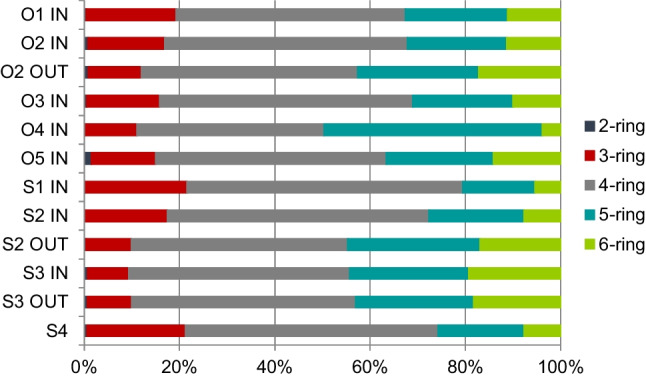
Distribution patterns of 2-, 3-, 4-, 5-, 6- ring PAHs in analysed sediment samples

Figure 2 presents the PAHs distribution patterns in sediment samples according to the number of rings in a molecule. The 2-to-3-ring, 4-ring, and 5-to-6-ring PAHs represent low, medium, and high molecular weight PAHs (LMW, MMW, and HMW PAHs), respectively (Amoako et al. 2011). PAHs with 4 rings were dominating in all sediment samples (with a content between 45 and 55%), followed by 5-ring PAHs (with the maximum of 5-rings PAHs obtained for O4 IN). The total percentage share of 3- and 6-rings PAHs was similar—between 15 and 20%. Moreover, an inverse relationship was observed for 3 and 6 rings PAHs: in samples with a higher percentage of 3 rings PAHs, the percentage of 6 rings PAHs was lower, and vice versa. In short, in most samples, the concentrations of HMW PAHs (4–6 rings) were higher than those of LMW PAHs (2–3 rings). LMW PAHs are produced mainly in automotive exhausts and by petroleum-derived fuels. Non-volatile PAHs (4–6 rings) are derived in relatively high amounts from high-temperature combustion processes (Dudhagara et al. 2016). The domination of HMW PAHs over LMW PAHs leads to the conclusion that combustion processes were the most significant source of PAHs in urban catchments. The dominance of HMW PAHs at most sites was probably due to their resistance to degradation and strong hydrophobicity. Because HMW PAHs have a higher molecular mass, they can accumulate more easily by adsorption on the OM of the sediments.

### PAH sources’ tracking

While understanding the sources of PAHs, it is essential to figure out the pathways whereby PAHs are released into the environment. Referring to the preliminary assessment based on HMW and LMW, it was assumed that the most probable source of PAHs is coal combustion followed by atmospheric deposition in the analysed areas. This finding is in agreement with results reported by Nawrot et al. (2020a) who investigated TM sources in the same urban area. It is worth highlighting here that in 2017, a regulation was introduced by the Minister of Development and Finance (2017) in Poland, which aimed to eliminate low-quality stoves. This was due to the large excess of the B(a)P pollution through so-called “low chimney emission” (< 40 m). The national report (2016) for the Pomeranian Voivodeship, including Gdansk, showed that B(a)P was exceeded in 2016 for PM10 (> 1 ng/m^3^). As mentioned before, due to atmospheric deposition PAHs released from coal burning processes enter water bodies and become deposited in sediments. In this study, the highest contamination with B(a)P was observed for sediments in O1 and O2 RTs, and in general, the concentration was higher for the Oliwski Stream catchment area. This observation corresponds very well to the old-type of heating systems predominantly used in this district—mainly solid fuel boilers, successively being replaced by gas boilers since 2017.

Another potential source may be surface runoff enriched with pollutants originating from transport (car exhaust fumes, wear of mechanical parts, and leakage of car fluids), which reaches the SDS and is delivered to urban streams and RTs. In order to confirm the above considerations on PAH sources, a set of tools was applied, including the diagnostic ratios method, statistical approach, and comparison of PAHs enriched sediments with TM concentration in bottom sediments of RTs.

#### PAHs’ ratios

**Fig. 3:**
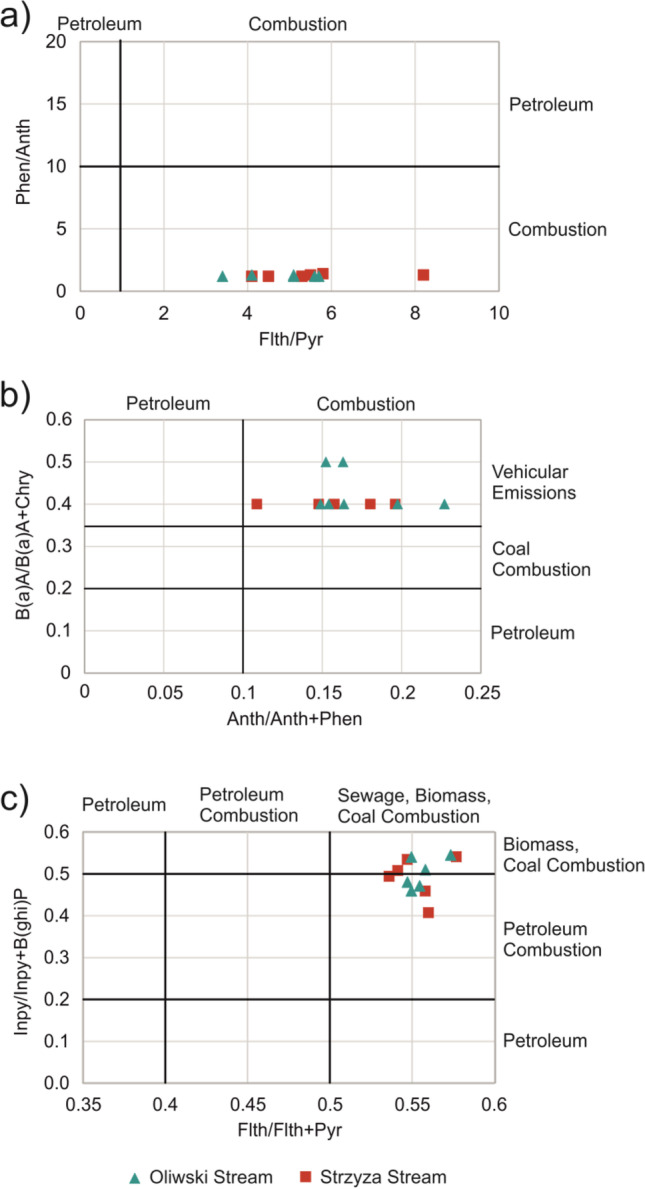
Isomeric ratios of **a**) Phen/Anth vs. Flth/Pyr **b**) B(a)A/B(a)A + Chry vs. Anth/Anth + Phen **c**) Inpy/Inpy + B(ghi)P vs. Flth/Flth + Pyr in the sediment samples collected from RTs

The origins of PAHs in sediment samples were tracked by means of calculating several PAH’ isomer ratios Phen/Anth vs. Flth/Pyr (Fig. 3a), B(a)A/B(a)A + Chry vs. Anth/Anth + Phen (Fig. 3b). The values of PAH ratios (Phen/Anth < 10, Flth/Pyr > 1, and B(a)A/B(a)A + Chry > 0.35, Anth/Anth + Phen > 0.1) confirm that most PAHs originate from pyrogenic sources. Due to the proximity of urbanized areas, the PAHs detected in the samples most likely come from “low chimneys” (old types of furnace), such as the combustion of solid fuel in boilers, wood in fireplaces, and petroleum in cars. The data presented by Crane et al. (2010) for Phen/Anth and Flth/Pyr ratios confirm that among the primary pyrogenic sources, diesel engine soot (Phen/Anth = 0.06; Flth/Pyr = 1.26), coal tar (Phen/Anth = 3.11; Flth/Pyr = 1.29), wood-burning emission (Phen/Anth = 6.41; Flth/Pyr = 1.26), and highway dust (Phen/Anth = 4.7; Flth/Pyr = 1.4) present similar ratios as those observed in this study. Moreover, the analyses performed by Sjögren et al. (1996) for heavy-duty diesel exhaust particles (*n* = 22) showed that Phen/Anth and Flth/Pyr ratios can be represented with a wide range of values such as 1.3–78 and 0.25–1.38, respectively.

Figure 3c presents the plots of Inpy/Inpy + B(ghi)P vs. Flth/Flth + Pyr. These ratios confirm mixed combustion sources such as biomass, coal, and petroleum combustion. PAHs from combustion processes were found near urbanised areas such as the coastal area of Palermo (Di Leonardo et al. 2009), the continental shelf of China (Liu et al. 2012), Delhi (Agarwal 2009) or Gdansk (Melnyk et al. 2015). Despite the close location of the RTs analysed to traffic arteries, petrogenic PAHs related to fuel leaks from cars were not detected. Proper maintenance and good technical state generally prevent vehicles from directly leaking fuel, unless some incidental traffic accidents occur.

#### Statistical approach

**Fig. 4:**
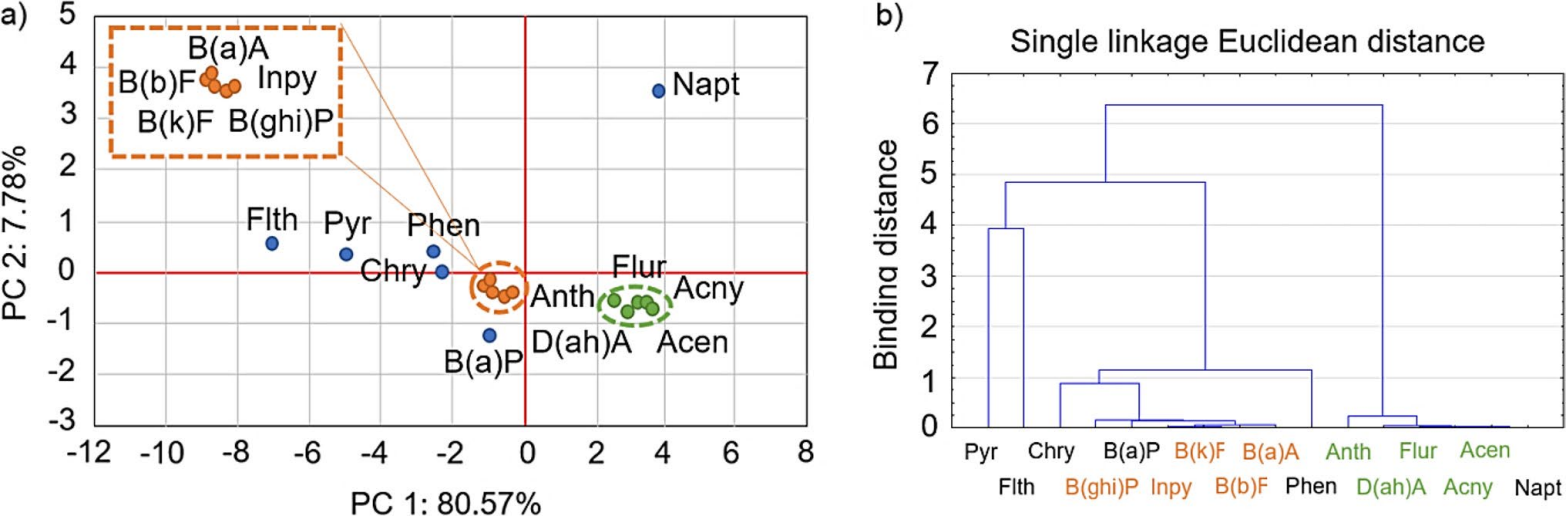
**a)** Principal components of 16 PAHs from RTs and **b**) hierarchical dendrogram for the classification of 16 PAHs based on their concentration in the sediments

Figure 4a shows the matrix of the individual PAHs in the principal components in the bottom sediment samples. The PCA analysis showed two main groups of variables. The percentage variance of PC1 and PC2 was 80.57% and 7.78% respectively, which in total accounted for 88.35% of the total variability. The first group (PC1) consists of 5- and 6-ring PAHs: B(a)A, Inpy, B(ghi)P, B(k)F, and B(a)A. Chry, B(a)P, Flth, and Pyr moderately load PC1. This group has significant positive loading with 4–6-ring PAHs. These species are associated with coal, grass, and wood combustion sources; therefore, PC1 can be categorised as a combustion source (Hezhong et al. 2016). PC1 is also moderately loaded by Phen, which is a 3-ring PAH. Phen is present in incomplete combustion and may come from vehicle emissions, coal and oil burning, wood combustion (Gad 2014), suggesting mixed sources. The second group (PC2) includes Flur, Acny, Acen, D(ah)A, and Anth, which are mostly 3-ring PAHs. Within PC2 the PAH concentrations were low (below 0.296 mg/kg d.w.). According to Soclo et al. (2000), such correlation or occurrence of PAHs may indicate the spill, volatilisation, or combustion of petroleum as an original source. Therefore, PC2 can be classified as petrogenic and low-temperature pyrogenic sources. Napt presents independent behaviour and, at the same time, the Napt concentration in sediment samples was low, which indicates that incomplete combustion-related sources of Napt can be excluded.

In this study, hierarchical cluster analysis (HCA) was also applied to identify the homogeneous groups of individual PAHs in the sediments of the RTs. According to the HCA results, 16 PAHs can be divided into three groups (Fig. 4b). These groups partially overlap with the groups established in the PCA. The first, smaller group, consists of Pyr and Flth, which are 4-ring PAHs mostly delivered from pyrogenic sources. The second group corresponds to PC1 with Chry, B(a)P, and Phen, which confirms that these PAHs originated from combustion and vehicular emission (mixed sources). The third group consists of 2- and 3-ring PAHs and one 5-ring hydrocarbon (D(ah)A). The main sources of PAHs in this group are spills, volatilisation, and petroleum combustion.

Incompletely burnt fuels are one of the sources of PAHs due to PAH compounds included in petroleum-based fuels. Frequently called fuel pyrolysis, this can probably be referred to as PAHs synthesis. For example, during this process, Acen heated to 700 °C under conditions precluding combustion, forms Pyr at a yield of 6.5% and some other PAHs, including Chry (Mullinger and Jenkins 2013). Both Pyr and Chry were found in high concentrations in analysed sediment samples, which means that such a process of PAHs synthesis was possible, emphasising the combustion sources of PAHs.

#### PAHs and TMs’ correlation

Comprehensive analyses of the sources of TMs for sediments collected from RTs from Grunwaldzka RT on the Oliwski Stream and Potokowa RT on the Strzyza Stream were carried out in our previous studies (Nawrot et al. 2020a; Szmagliński et al. 2021; Wojciechowska et al. 2019). The analyses focused on the effects of RDS and the tyre wear of the waste parts of vehicles on the contamination of the bottom sediments. In this context, the main sources found in TM analyses pointed out the coal combustion as a source of Pb. In Grunwaldzka RT Cd, Ni, and Cr were evaluated as being emitted as a result of car parts wear and tear, Cu possibly originated from vehicle use or run-off from roofs covered with copper sheeting, while Zn was evaluated as a result of tyre wear. In Potokowa RT, Zn, Pb, Ni, Cr, and Fe were identified as delivered from mixed pathways of traffic and coal combustion. Cd with, in general, low concentrations was connected with geochemical background, while Cu was slightly correlated with Zn, Pb, Ni, Cr, and Fe presenting quasi-independent behaviour. The summary of the results of metal concentrations in the sediments in the analysed period is presented in Tab.S.5.

Here, to further confirm and verify the PAHs sources, we tested the relationships between PAHs and TMs using FA. Table 2 explores the factor loadings for PAHs and TMs in sediments collected from RTs. Analyses were performed for data obtained for the Oliwski and Strzyza streams separately. In the case of the Oliwski Stream, the FA reduces the number of variables to three factors (F1, F2, and F3), and to two factors for the Strzyza Stream (F1, F2), which explain in total 93.03% and 92.66% of the data variance, respectively. F1 in the Oliwski Stream is heavily loaded with all PAHs. Zn and Fe accounted for 68.86% of the total variance. This factor sheds new light on the potential source of contained elements. F1 presents a strong PAH correlation with Zn and Fe, which can be found as compounds of passenger car tyre tread (Thorpe and Harrison 2008) in amounts up to 10,250 mg/kg of Zn (Legret and Pagotto 1999) and 4,600 mg/kg of Fe (Hildemann et al. 1991), that PAH emission (bound in F1) emission is connected with tyre wear. Aatmeeyata and Sharma (2010) confirmed that PAHs emitted from tyre were with the emission factor established at the level of 378 ng/tyre/km. Among all PAHs Pyr followed by B(ghi)P was noted at the highest concentrations of 30 ± 4 mg/kg and 17 ± 2 mg/kg, respectively (Aatmeeyata and Sharma 2010). F2 and F3 accounted for 14.06% and 10.29% of the total variance corresponding to strong relationships between TMs. F1 in the Strzyza Stream is heavily loaded with all PAHs, Zn, Cu, Cd, Ni, Cr, and Fe, again demonstrating mixed sources of coal combustion and emission from vehicle sources (including tyre wear). F1 accounted for 85.52% of the total variance. According to Thorpe and Harrison (2008), car brake lining and car brake dust cause significant emissions of TMs. In the case of the car brake lining, the maximum documented emission was as follows (in mg/kg) and decreased in the following order: Cu (234,000) > Zn (188,000) > Pb (119,000) > Fe (63,700) > Ni (660) > Cr (411) > Cd (41.4). At the same time, car brake dust emission was (in mg/kg): Fe (53,700) > Cu (39,400) > Zn (27,300) > Cr (1,320) > Pb (1,290) > Ni (730) > Cd (2.6). F2 for the Strzyza Stream represented 7.14% of the total variance with a high load of Pb (0.93). This independent feature of Pb could be explained by the behaviour of Pb that is governed by both fuel characteristics and operating conditions. It is well documented that even moderate changes in fuel composition and/or experimental conditions might lead to different results of Pb concentrations, which is reflected by not fully consistent conclusions found in different literature studies (Bartoňová et al. 2019).

**Table 2:** Factor analysis (FA) indicating the factor loading (F1–F2) for polycyclic aromatic hydrocarbons (PAHs) and trace metals (TMs) in sediments collected from retention tanks (RTs) on the Oliwski and Strzyza streams

Components	Factors				
	Oliwski Stream			Strzyza Stream	
	F1	F2	F3	F1	F2
Zn	** − 0.75**	** − **0.60	0.28	** − 0.96**	0.09
Cu	0.17	** − **0.46	** − 0.85**	** − 0.90**	0.09
Pb	0.01	** − **0.68	** − 0.69**	0.00	**0.93**
Cd	0.07	**0.95**	** − **0.19	** − 0.92**	0.34
Ni	0.35	**0.88**	** − **0.32	** − 0.97**	** − **0.09
Cr	0.43	0.20	** − 0.86**	** − 0.93**	** − **0.11
Fe	** − 0.68**	0.59	** − **0.15	** − 0.70**	0.45
Napt	** − 0.87**	** − **0.15	** − **0.18	** − 0.97**	** − **0.20
Acny	** − 0.94**	0.09	** − **0.16	** − 0.98**	0.06
Acen	** − 0.82**	0.16	0.09	** − 0.61**	** − **0.46
Flur	** − 0.94**	0.12	0.04	** − 0.93**	** − **0.34
Phen	** − 0.93**	0.14	0.04	** − 0.97**	** − **0.05
Anth	** − 0.97**	0.08	0.02	** − 0.98**	** − **0.12
Flth	** − 0.99**	0.07	** − **0.07	** − 1.00**	** − **0.03
Pyr	** − 0.99**	0.09	** − **0.04	** − 0.99**	** − **0.08
B(a)A	** − 0.99**	0.06	** − **0.05	** − 0.99**	0.07
Chry	** − 0.99**	0.03	** − **0.09	** − 0.99**	0.06
B(b)F	** − 0.98**	** − **0.02	** − **0.12	** − 0.99**	0.05
B(k)F	** − 1.00**	0.05	** − **0.07	** − 0.98**	0.11
B(a)P	** − 0.90**	** − **0.13	0.18	** − 0.99**	0.09
Inpy	** − 0.95**	** − **0.06	** − **0.13	** − 0.98**	0.04
A(ah)A	** − 0.98**	** − **0.04	** − **0.09	** − 0.98**	0.02
B(ghi)P	** − 0.96**	** − **0.05	** − **0.14	** − 0.98**	** − **0.03
Eigenvalue	15.84	3.23	2.37	20.64	1.64
% of variance	68.86	14.06	10.29	85.52	7.14
% of cumulative	68.86	82.74	93.03	85.52	92.66

**Bold** – correlated elements inside a factor.

In summary, the major source of PAHs in surface sediments was determined to be coal and petroleum combustion due to traffic loads and heating systems. The synergy observed in the sources analyses of PAHs and TMs confirms relatively convergent pollution sources. An additional source of PAHs correlated with TMs is related to the wearing of consumable parts of the vehicles, such as tyre wear, car brake lining, and car brake dust.

### Toxicity to humans

Toxic equivalency quantities (TEQs) calculated in reference to toxic equivalency factors (TEFs) are given in Tab.S.6. TEQ > 1 was calculated for 2 sampling sites in reference to B(a)P (O1 IN—1.37 mg/kg d.w. and O2 IN—1.27 mg/kg d.w.). Meanwhile, TEQs for Σ16PAHs in these samples were (in mg/kg d.w.) 2.08 and 2.02, respectively. The TEQs calculated for the sum of Σ7CPAHs for these samples constitute 99% of the TEQs calculated for Σ16PAHs, which further raising concerns about sediment toxicity. Moreover, B(a)P constitutes 58–96% of Σ16PAHs, thus it seems to be responsible for most of the toxicological risk identified for the studied sediments. Similar results can be found elsewhere (Yuan et al. 2021 and Gdara et al. 2017), highlighting the importance of using B(a)P as an indicator of carcinogenic PAHs that affect sediments.

### Ecotoxicological effect

#### SQGs

Our results were compared with TEC and PEC values determined for 10 PAHs (Napt, Flur, Phen, Anth, Flth, Pyr, B(a)A, Chry, B(a)P, D(ah)A) and ∑PAHs (MacDonald et al. 2000). Based on the threshold levels presented in the SQGs for PAHs detected in freshwater sediments (MacDonald et al., 2000), the concentrations of Napt were below TEC in all sediments analysed (Tab.1). Furthermore, almost all PAH concentrations were below TEC at sampling points S1 IN, S2 IN, S4 IN, and O4 OUT, indicating that negative biological effects on organisms at those sampling points are expected to be rare. At the other sampling points, most PAHs have values between TEC and PEC, indicating that harmful effects on the biota can occur occasionally. Negative biological effects can occur frequently at the O2 OUT, and O1 OUT where almost all PAH exceed the PEC values (Table 1).

#### RQ risk

Tab.S.7 report the mean values of RQ_(NCs)_ and RQ_(MPCs)_ for sediments collected from the Oliwski and Strzyza streams. The mean values of RQ_(NCs)_ of almost all PAHs (with some exception for D(ah)A, B(ghi)P, Chry, B(a)P, Inpy) were above unity (> 1), which indicates moderate ecological risk. The highest RQ_(NCs)_ was observed in case of Pyr (4-ring PAH), which highlights its main influence on the increase of overall ecological risk in the tested sediments. In general, the proportion of RQ_(NCs)_ for LMW to HMW fell within the range of 5:1 to 13:1, indicating the important proportion of less mutagenic, low, and moderate molecular PAHs. At the same time, LMW PAHs pose much more risk than HMW PAHs in sediments collected from urban RTs (especially in the Oliwski Stream). The high risk associated with RQ_(MPCs)_ value (≥ 1) was obtained for Acen, Flur, Phen, Anth, Flth, Pyr, B(a)A, and B(b)F in O1, Napt, Flur, Phen, Anth, Flth, Pyr, B(a)A, and B(b)F in O2 IN, and Phen, Anth, Pyr, B(a)A in O2 OUT, as well as for Pyr in O4 and O5. In the case of the Strzyza Stream RT’ greater RQ_(MPCs)_ than unity (≥ 1) was observed for Pyr in S2, S3, and for B(a)A and B(b)F in S3. The RQ for the sum of PAHs (Tab.3) determined with reference to NCs and MPCs is at a high risk level for almost all sampling points. At least moderate risk (1^st^ level) is assessed for bottom sediments deposited in RTs in the Strzyza Stream and at least moderate risk (2^nd^ level) for those in the Oliwski Stream. Aquatic environments with high concentrations of PAHs can pose a potential ecological risk, and concerted efforts are required to ensure their protection. In the case of Gdansk, RTs are widely used for fishing; however, due to the obtained high ecological risk levels, the survival of selected species (especially those that move along the bottom and are exposed to direct contact with sediments) may be limited (Table 3).

**Table 3:**
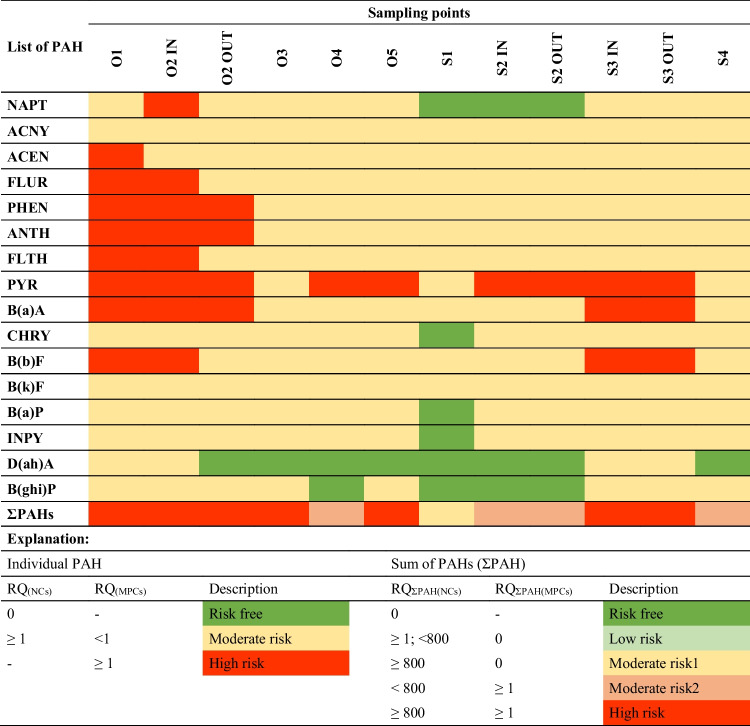
Risk assessment of sediments samples collected from the Oliwski (O1–O5) and Strzyza (S1–S4) streams based on RQ_(NCs)_ and RQ_(MPCs)_ values

## Conclusions

In this study, a comprehensive survey of 16 PAHs was conducted in sediments deposited in urban RTs. The receivers of SDS are exposed to the accumulation of potentially toxic compounds and thus the deposited sediments present a fingerprint of processes taking place within the catchment area. The sum of Σ16PAHs (in mg/kg d.w.) in bottom sediments samples from investigated urban catchments ranged from 1.95 ± 0.64 to 20.4 ± 6.8 for RTs located on the Oliwski Stream and from 0.50 ± 0.17 to 8.6 ± 2.9 for RTs located on the Strzyza Stream. The highest contamination of the bottom sediments with PAHs was observed in O1 and O2 of the Oliwski Stream (20.4 ± 6.8 and 16.9 ± 5.7 mg/kg d.w., respectively). In most samples, the concentrations of non-volatile HMW PAHs (4–6 rings) were higher than LMW PAHs (2–3 rings), which suggested that they came from high-temperature combustion processes.

The implemented PAHs isomer ratios Phen/Anth, Flth/Pyr, B(a)A/B(a)A + Chry, Inpy/Inpy + B(ghi)P, Flth/Flth + Pyr, Anth/Anth + Phen, Flth/Flth + Pyr indicated biomass, coal, and petroleum combustion delivery pathways. Petrogenic PAHs related to fuel leaks from cars were not detected. Statistical analyses gave generally coherent conclusions regarding PAH sources. Coal and petroleum combustion related to heating systems and traffic loads were the most common. The FA performed for PAHs and TMs bound in sediments indicated vehicles wear and tears such as tyre wear, car brake lining, and car brake dust.

TEQ for carcinogenic PAHs (7CPAHs) indicated that the ecological risk of multiple B(a)P was quite high at the O1 and O2 sampling sites on the Oliwski Stream. Based on SQG at O2 OUT and O1 OUT sampling points, detected concentrations of PAHs may present a high risk of adverse effects in organisms. The RQ of individual PAHs and Σ16PAHs presented a moderate and high risk for biota.

The general conclusion of this study points to the raised pollution levels of PAHs in bottom sediments deposited in RTs on urban streams. PAHs content in sediments reflect pollution associated with anthropogenic activity in the urban area. So, the results of this study may be considered on two levels. The first is a direct assessment of sediment status, while the second is an indirect indicator of environmental pollution existing in the area. Taking into account the latter, the results of this study point out the need to monitor and counteract pollution in the Gdansk urban area. In the region analysed, combustion processes associated with heating systems and transport were identified as the main contributors of PAHs, while the main pathway of pollutants to deposited sediments is atmospheric deposition and stormwater runoff. This indirectly shows that atmospheric pollution is a major problem that can result in lower living standards and exposure to health risks for the rapidly increasing population of Gdansk. These findings are consistent with reports of environmental agencies responsible for monitoring atmospheric pollution. Further research directions can focus on explaining the mutual relationship between air pollution, sediments, and human health. Furthermore, future increasing the number of screened retention reservoirs in the Gdansk area, and thus analysing a larger data sets of contaminant concentrations, what would allow to identify the most exposed areas, as well as the implementation of protective measures. Pro-ecological activities in the context of planned mitigation strategies for air pollution should aim, inter alia, at decreasing fuel consumption, introducing sustainable transport, and the use of renewable energy sources instead of burning fossil fuels. The results of this study can serve as a reference for future research to monitor potential change of pollution status in the context of promoting renewable energy consumption and electrical cars, and on the other hand, to monitor the environmental effects of energy crisis in 2022 that may lead to increased consumption of low quality solid fuels.

## Supplementary Information

Below is the link to the electronic supplementary material.

Supplementary file1 (DOCX 67 KB)

## Data Availability

The authors confirm that the data supporting the findings of this study are available within the article and its supplementary material.

## Abbreviations

Σ7CPAHs: Cancerogenic PAH concentration
Acen: Acenaphthene
Acny: Acenaphthylene
Anth: Anthracene
B(a)A: Benzo(a)anthracene
B(a)P: Benzo(a)pyrene
B(b)F: Benzo(b)fluoranthene
B(ghi)P: Benzo(g,h,i)perylene
B(k)F: Benzo(k)fluoranthene
CEQ: Cumulative health hazard
Chry: Chrysene
D(ah)A: Dibenzo(a,h)anthracene
FA: Factor analysis
Flth: Fluoranthene
Flur: Fluorene
HCA: Hierarchical cluster analysis
HMW: High molecular weight
Inpy: Indeno(1,2,3-cd)pyrene
Log Kow: The log octanol/water partition coefficient
LMW: Low molecular weight
MEQ: Mutagenic equivalent
MMW: Medium molecular weight
MPCs: Maximum permissible concentrations
Napt: Naphthalene
NCs: Negligible concentrations
PAHs: Polycyclic aromatic hydrocarbons
PC: Principal component
PCA: Principal component analysis
PEC: Probable effect concentration
Phen: Phenanthrene
Pyr: Pyrene
RQ: Risk quotient
RTs: Retention tanks
SDS: Stormwater drainage system
TEC: Threshold effect concentration
TEF: Toxicity equivalent factor
TEQ: Toxic equivalent quotient
