# Supplementary material for: A multi-criteria approach to investigate spatial distribution, sources, and the potential toxicological effect of polycyclic aromatic hydrocarbons (PAHs) in sediments of urban retention tanks
Source: Environ Sci Pollut Res Int. 2022 Nov 17;30(10):27895–911. doi: 10.1007/s11356-022-24168-1 (PMC9995424; doi:10.1007/s11356-022-24168-1)
Supplement: Supplementary file 1 — Supplementary file1 (DOCX 67 KB) [file 11356_2022_24168_MOESM1_ESM.docx]

**Supplementary material** to the manuscript entitled

**A multi-criteria approach to investigate spatial distribution, sources, and the potential toxicological effect of polycyclic aromatic hydrocarbons (PAHs) in sediments of urban retention tanks**

Nicole Nawrot*^1^, Anna Pouch**^1^, Karolina Matej-Łukowicz*, Ksenia Pazdro**, Muhammad Mohsin***, Shahabaldin Rezania****, Ewa Wojciechowska*

* Gdansk University of Technology, Faculty of Civil and Environmental Engineering, Narutowicza 11/12, 80-233, Gdansk, Poland

** Institute of Oceanology of the Polish Academy of Sciences, Marine Geotoxicology Laboratory, Powstańców Warszawy 55, 81-712 Sopot, Poland

*** School of Forest Sciences, University of Eastern Finland, Yliopistokatu 7, P.O. Box 111, 80100 Joensuu, Finland

**** Department of Environment and Energy, Sejong University, Seoul, 05006, South Korea

^1^ N.N. and A.P. contributed equally to this work

**Table Supplement 1** Site, retention tanks’ area, capacity, damming level (normal, maximum), grain size, and organic matter (OM) content in sediments of analysed retention tanks (RTs) (http://www.gdmel.pl)

| **No.** | **Site (RT)** | **^*^km of stream [km+m]** | **Area [ha]** | **Retention Capacity [m^3^]** | **Adjacent area characteristic** | **Damming level  [m a.s.l.]** | | **Grain size [%]** | | | | | | **Organic matter [%]** |
| --- | --- | --- | --- | --- | --- | --- | --- | --- | --- | --- | --- | --- | --- | --- |
|  |  |  |  |  |  | **normal** | **max** | **<0.063** | **>0.063** | **>0.125** | **>0.250** | **>0.500** | **>1.0** |  |
| **Oliwski Stream** | | | | | | | | | | | | | | |
| O1 | Spacerowa IN | 4+010 | 1.08 | 5040 | paved surfaces; main stream of vehicle traffic; stormwater outlets directly to the RT; | 33.4 | 33.8 | 24.8 | 24.8 | 21.5 | 13.2 | 8.4 | 4.18 | 21.2 |
| O2 | Grunwaldzka IN | 2+143 | 1.69 | 8450 |  | 20.4 | 20.9 | 2.68 | 0.21 | 34.1 | 44.0 | 11.4 | 3.81 | 15.0 |
|  | Grunwaldzka OUT |  |  |  |  |  |  | 25.3 | 22.9 | 21.7 | 14.5 | 4.43 | 5.95 | 18.9 |
| O3 | Subislawa IN | 2+411 | 3.1 | 15500 | soft (green) surfaces; residential district | 15.7 | 16.2 | 7.18 | 11.2 | 29.7 | 31.5 | 12.4 | 4.66 | 8.21 |
| O4 | Chlopska IN | 1+411 | 1.2 | 6000 |  | 9.0 | 9.5 | 3.88 | 9.18 | 37.8 | 32.5 | 8.89 | 4.20 | 5.14 |
| O5 | Orlowska IN | 0+920 | 0.58 | 2900 |  | 5.60 | 6.10 | 2.83 | 7.86 | 33.9 | 45.4 | 5.82 | 2.22 | 7.11 |
| **Strzyza Stream** | | | | | | | | | | | | | | |
| S1 | Nowiec IN | 7+485 | 0.52 | 8336 | soft (green) surfaces; single-family housing; | 64.41 | 66.02 | 1.51 | 2.33 | 21.9 | 41.7 | 26.6 | 5.35 | 2.89 |
| S2 | Ogrodowa IN | 5+995 | 0.30 | 1500 | main stream of vehicle traffic | 46.5 | 47.0 | 27.9 | 38.6 | 13.2 | 10.9 | 7.00 | 2.50 | 13.8 |
|  | Ogrodowa OUT |  |  |  |  |  |  | 40.2 | 27.4 | 14.3 | 8.6 | 6.19 | 2.84 | 15.4 |
| S3 | Potokowa IN | 5+450 | 0.30 | 6700 | main stream of vehicle traffic; stormwater outlets directly to the RT | 39.1 | 40.0 | 27.4 | 23.4 | 23.1 | 13.8 | 9.32 | 2.83 | 14.1 |
|  | Potokowa OUT |  |  |  |  |  |  | 22.1 | 32.6 | 16.8 | 13.6 | 11.1 | 2.85 | 12.4 |
| S4 | Srebrniki IN | 4+730 | 2.58 | 64500 | main stream of vehicle traffic | 36.82 | 38.0 | 16.6 | 19.1 | 15.6 | 15.8 | 14.7 | 10.5 | 5.41 |
| * IN – inflow; OUT - outflow | | | | | | | | | | | | | | |

**Table Supplement 2** Limits of detection [mg/kg d.w.], limits of quantification [mg/kg d.w.] and recoveries [%] of individual PAHs.

|  | **Limit of detection in mg/kg d.w.** | **Limit of quantification  in mg/kg d.w.** | **Recovery * in %** |
| --- | --- | --- | --- |
| **Napt** | 0.0003 | 0.001 | 67.89 |
| **Acny** | 0.0003 | 0.001 | 87.16 |
| **Acen** | 0.0003 | 0.001 | 61.79 |
| **Flur** | 0.0003 | 0.001 | 83.70 |
| **Phen** | 0.0003 | 0.001 | 95.70 |
| **Anth** | 0.0003 | 0.001 | 82.77 |
| **Flth** | 0.0003 | 0.001 | 82.54 |
| **Pyr** | 0.0003 | 0.001 | 91.50 |
| **B(a)A** | 0.0003 | 0.001 | 91.50 |
| **Chry** | 0.0003 | 0.001 | 92.78 |
| **B(b)F** | 0.0003 | 0.001 | 110.36 |
| **B(k)F** | 0.0003 | 0.001 | 97.05 |
| **B(a)P** | 0.0003 | 0.001 | 112.61 |
| **Inpy** | 0.0003 | 0.001 | 116.73 |
| **D(a,h)A** | 0.0003 | 0.001 | 114.21 |
| **B(g,h,i)P** | 0.0003 | 0.001 | 109.80 |

*The reference material, LGC6188 - River Sediment from LGC (Teddington, United Kingdom), was used for quality control of PAHs.

**Table Supplement 3** Concentration of PAHs [mg/kg d.w.] ± SD (n=3) in urban streams (Oliwski and Strzyza) sediments in Gdansk, Poland

| **PAH** | **PAHs concentration in mg/kg d.w. in sediment samples of analyzed retention tanks** | | | | | | | | | | | | | | | | | | | | | | | | | | | | | | | | | | | |
| --- | --- | --- | --- | --- | --- | --- | --- | --- | --- | --- | --- | --- | --- | --- | --- | --- | --- | --- | --- | --- | --- | --- | --- | --- | --- | --- | --- | --- | --- | --- | --- | --- | --- | --- | --- | --- |
|  | **Oliwski Stream** | | | | | | | | | | | | | | | | | | **Strzyza Stream** | | | | | | | | | | | | | | | | | |
|  | **O1 IN** | | | **O2 IN** | | | **O2 OUT** | | | **O3 IN** | | | **O4 IN** | | | **O5 IN** | | | **S1 IN** | | | **S2 IN** | | | **S2 OUT** | | | **S3 IN** | | | **S3 OUT** | | | **S4 IN** | | |
| **Napt** | 0.092 | ± | 0.028 | 0.15 | ± | 0.045 | 0.039 | ± | 0.011 | 0.02 | ± | 0.006 | 0.004 | ± | 0.001 | 0.049 | ± | 0.015 | p.0.001 | | | p.0.001 | | | 0.013 | ± | 0.004 | 0.063 | ± | 0.019 | 0.048 | ± | 0.014 | 0.01 | ± | 0.003 |
| **Acny** | 0.06 | ± | 0.018 | 0.076 | ± | 0.023 | 0.017 | ± | 0.005 | 0.023 | ± | 0.007 | 0.004 | ± | 0.001 | 0.017 | ± | 0.005 | 0.002 | ± | 0.001 | 0.006 | ± | 0.001 | 0.021 | ± | 0.006 | 0.04 | ± | 0.012 | 0.032 | ± | 0.010 | 0.01 | ± | 0.002 |
| **Acen** | 0.229 | ± | 0.069 | 0.057 | ± | 0.017 | 0.048 | ± | 0.014 | 0.024 | ± | 0.007 | 0.009 | ± | 0.003 | 0.021 | ± | 0.006 | 0.005 | ± | 0.001 | 0.017 | ± | 0.005 | 0.011 | ± | 0.003 | 0.03 | ± | 0.009 | 0.024 | ± | 0.007 | 0.03 | ± | 0.008 |
| **Flur** | 0.248 | ± | 0.074 | 0.125 | ± | 0.037 | 0.055 | ± | 0.016 | 0.037 | ± | 0.011 | 0.014 | ± | 0.004 | 0.038 | ± | 0.011 | 0.009 | ± | 0.002 | 0.019 | ± | 0.006 | 0.018 | ± | 0.005 | 0.072 | ± | 0.021 | 0.059 | ± | 0.018 | 0.04 | ± | 0.01 |
| **Phen** | 2.76 | ± | 0.83 | 1.3 | ± | 0.39 | 0.59 | ± | 0.18 | 0.4 | ± | 0.12 | 0.143 | ± | 0.043 | 0.323 | ± | 0.097 | 0.082 | ± | 0.024 | 0.187 | ± | 0.056 | 0.271 | ± | 0.081 | 0.48 | ± | 0.14 | 0.45 | ± | 0.14 | 0.27 | ± | 0.08 |
| **Anth** | 0.54 | ± | 0.16 | 0.32 | ± | 0.096 | 0.115 | ± | 0.035 | 0.07 | ± | 0.021 | 0.042 | ± | 0.013 | 0.058 | ± | 0.017 | 0.01 | ± | 0.003 | 0.035 | ± | 0.011 | 0.047 | ± | 0.014 | 0.117 | ± | 0.034 | 0.099 | ± | 0.030 | 0.05 | ± | 0.014 |
| **Flth** | 3.6 | ± | 1.1 | 2.81 | ± | 0.84 | 0.96 | ± | 0.29 | 0.71 | ± | 0.21 | 0.277 | ± | 0.083 | 0.58 | ± | 0.17 | 0.112 | ± | 0.033 | 0.296 | ± | 0.089 | 0.6 | ± | 0.18 | 1.41 | ± | 0.42 | 1.18 | ± | 0.36 | 0.38 | ± | 0.11 |
| **Pyr** | 2.95 | ± | 0.88 | 2.09 | ± | 0.63 | 0.76 | ± | 0.23 | 0.57 | ± | 0.17 | 0.227 | ± | 0.068 | 0.48 | ± | 0.14 | 0.088 | ± | 0.026 | 0.245 | ± | 0.073 | 0.44 | ± | 0.13 | 1.22 | ± | 0.37 | 1.00 | ± | 0.30 | 0.30 | ± | 0.09 |
| **B(a)A** | 1.53 | ± | 0.61 | 1.19 | ± | 0.45 | 0.39 | ± | 0.15 | 0.28 | ± | 0.11 | 0.119 | ± | 0.048 | 0.235 | ± | 0.094 | 0.038 | ± | 0.015 | 0.125 | ± | 0.049 | 0.29 | ± | 0.12 | 0.54 | ± | 0.21 | 0.45 | ± | 0.18 | 0.15 | ± | 0.059 |
| **Chry** | 1.73 | ± | 0.52 | 1.57 | ± | 0.47 | 0.49 | ± | 0.15 | 0.36 | ± | 0.1 | 0.144 | ± | 0.043 | 0.34 | ± | 0.1 | 0.054 | ± | 0.016 | 0.165 | ± | 0.049 | 0.41 | ± | 0.12 | 0.82 | ± | 0.25 | 0.72 | ± | 0.21 | 0.18 | ± | 0.055 |
| **B(b)F** | 1.34 | ± | 0.4 | 1.44 | ± | 0.43 | 0.36 | ± | 0.11 | 0.224 | ± | 0.067 | 0.076 | ± | 0.022 | 0.251 | ± | 0.075 | 0.023 | ± | 0.007 | 0.09 | ± | 0.027 | 0.34 | ± | 0.1 | 0.75 | ± | 0.22 | 0.62 | ± | 0.18 | 0.11 | ± | 0.032 |
| **B(k)F** | 1.42 | ± | 0.42 | 1.28 | ± | 0.38 | 0.34 | ± | 0.1 | 0.279 | ± | 0.083 | 0.092 | ± | 0.027 | 0.266 | ± | 0.079 | 0.03 | ± | 0.009 | 0.112 | ± | 0.033 | 0.36 | ± | 0.1 | 0.66 | ± | 0.19 | 0.54 | ± | 0.16 | 0.12 | ± | 0.037 |
| **B(a)P** | 1.37 | ± | 0.41 | 1.27 | ± | 0.38 | 0.317 | ± | 0.095 | 0.229 | ± | 0.069 | 0.72 | ± | 0.21 | 0.207 | ± | 0.062 | 0.022 | ± | 0.007 | 0.09 | ± | 0.027 | 0.302 | ± | 0.09 | 0.57 | ± | 0.17 | 0.48 | ± | 0.14 | 0.11 | ± | 0.032 |
| **Inpy** | 1.22 | ± | 0.36 | 1.58 | ± | 0.47 | 0.295 | ± | 0.088 | 0.171 | ± | 0.051 | 0.034 | ± | 0.01 | 0.229 | ± | 0.069 | 0.011 | ± | 0.003 | 0.062 | ± | 0.018 | 0.35 | ± | 0.1 | 0.82 | ± | 0.25 | 0.66 | ± | 0.20 | 0.07 | ± | 0.02 |
| **D(ah)A** | 0.257 | ± | 0.077 | 0.296 | ± | 0.089 | 0.049 | ± | 0.015 | 0.029 | ± | 0.009 | 0.007 | ± | 0.002 | 0.035 | ± | 0.01 | 0.002 | ± | 0.001 | 0.011 | ± | 0.003 | 0.066 | ± | 0.02 | 0.168 | ± | 0.05 | 0.13 | ± | 0.04 | 0.01 | ± | 0.003 |
| **B(ghi)P** | 1.04 | ± | 0.31 | 1.32 | ± | 0.4 | 0.284 | ± | 0.085 | 0.192 | ± | 0.058 | 0.04 | ± | 0.012 | 0.248 | ± | 0.074 | 0.016 | ± | 0.005 | 0.054 | ± | 0.016 | 0.297 | ± | 0.089 | 0.84 | ± | 0.25 | 0.64 | ± | 0.19 | 0.08 | ± | 0.023 |
| **Sum** | 20.4 | ± | 6.8 | 16.9 | ± | 5.7 | 5.1 | ± | 1.7 | 3.6 | ± | 1.2 | 1.95 | ± | 0.65 | 3.4 | ± | 1.1 | 0.5 | ± | 0.17 | 1.52 | ± | 0.51 | 3.8 | ± | 1.3 | 8.6 | ± | 2.9 | 7.1 | ± | 2.4 | 1.91 | ± | 0.64 |

**Table Supplement 4** PAH in freshwater and marine sediments in the Pomeranian area – literature data

| **Area** | **Sampling year** | **Number of PAHs analysed** | **∑12 PAH (mg/kg d.w.)** | **References** |
| --- | --- | --- | --- | --- |
| Radunskie lake | 2012 | 12 | 0.035 – 2.408 | Czarnecka 2012 |
| Klasztorne Male lake | 2011 | 12 | 15.658 (median) | Tylman et al. 2011 |
| Druzno lake | 2016 | 17 | 1.528 | CIEP, 2017 |
| Ostrowickie lake | 2018-2019 | 17 | 18,353 | CIEP, 2019 |
| Czluchowskie lake |  |  | 137,200 |  |
| Baltic Sea, Gulf of Gdańsk (coastal zone) | 2003 - 2007 | 12 | 0.009 – 5.100 | Lubecki & Kowalewska, 2010 |

Czarnecka K., 2012. Geochemical record of environmental changes in upper bottom sediments of the Upper Radun Lake. [Geochemiczny zapis zmian środowiska w stropowych osadach dennych jeziora raduńskiego górnego] PhD thesis, in polish, University of Gdansk, 126pp.

CIEP, 2017. Test results and comprehensive assessment of the state of bottom sediments of rivers and lakes in 2016-2017. Comparison with the results from 2010-2015. Chief Inspectorate of Environmental Protection [Wyniki badań i ocena kompleksowa stanu osadów dennych rzek i jezior w latach 2016-2017. Porównanie z wynikami z lat 2010-2015. Główny Inspektorat Ochrony Środowiska], Warsaw, Poland, in polish

CIEP, 2019. Monitoring of bottom sediments of rivers and lakes in 2018-2019. "The state of contamination of bottom sediments of rivers and lakes in 2018." Chief Inspectorate of Environmental Protection [Monitoring osadów dennych rzek i jezior w latach 2018 - 2019. Raport pt. „Stan zanieczyszczenia osadów dennych rzek i jezior w 2018 roku.” Główny Inspektorat Ochrony Środowiska], Warsaw, Poland, in polish

Lubecki, L., Kowalewska, G., 2010. Distribution and fate of polycyclic aromatic hydrocarbons (PAHs) in recent sediments from the Gulf of Gdańsk (SE Baltic)* Sediment pollution Sediments Hydrocarbons Risk assessment PAHs Coastal zone Baltic Gulf of Gdańsk. Oceanologia 52, 669–703.

Tylmann W., Bełdowski J., Borowiak D., Fedorowicz S., Jańczak J., Kozłowska K., Pazdro K., Walkusz-Miotk J., Weisbrodt D., 2013. Inwentaryzacja ładunku zanieczyszczeń skumulowanych w osadach dennych jezior kartuskich jako szansa na ich skuteczną rekultywację. Raport końcowy z realizacji projektu WFOŚ/D/210/141/2011

**Table Supplement 5** Average concentration of TMs [mg/kg d.w.] (n=3) in urban streams (Oliwski and Strzyza) sediments in Gdansk, Poland

| **Sampling point** | | **Trace metal concentration in mg/kg d.w.** | | | | | | |
| --- | --- | --- | --- | --- | --- | --- | --- | --- |
|  |  | **Zn** | **Cu** | **Pb** | **Cd** | **Ni** | **Cr** | **Fe** |
| **Oliwski Stream** | | | | | | | | |
| O1 | OUT | 51.66 | 12.76 | 10.27 | 0.09 | 4.11 | 12.1 | 22,142 |
| O2 | IN | 59.8 | 4.72 | 14.7 | 0.121 | 3.56 | 14.7 | 10,682 |
| O2 | OUT | 3.4 | 9.01 | 2.04 | **0.311** | **12.3** | 24.3 | 26,063 |
| O3 | IN | **82.71** | 22.29 | 22.21 | 0.14 | 3.86 | 16.7 | 24,560 |
| O4 | IN | 44.2 | **56.7** | **45.8** | 0.115 | 5.2 | **26.8** | 18,569 |
| O5 | IN | **82.6** | 2.88 | 11.4 | 0.167 | 4.3 | 11.7 | **30,862** |
| **Strzyża Stream** | | | | | | | | |
| S1 | IN | 13.6* | 3.24* | 4.91* | 0.020* | 2.55* | 10.9* | 3,993* |
| S2 | IN | 121* | 23.1* | **217*** | 0.178* | 5.41* | 2.45* | 26,391* |
| S2 | OUT | 228* | 44.4* | **309*** | 0.307* | 5.8* | 12.4* | 31,150* |
| **S3** | **IN** | **584*** | **119*** | 81.1* | 0.388* | **25.8*** | 58.7* | 25,401* |
| **S3** | **OUT** | **791*** | **216*** | 87* | **0.552*** | **23.4*** | **74.5*** | **50,480*** |
| S4 | IN | 53.6* | 13.4* | 25.1* | 0.101* | 2.58* | 6.89* | 21,411* |
| Local geochemical background | | 26.2* | 6.07* | 10.1* | 0.06* | 4.86* | 5.06* | 9,731* |
| **Bold** – the highest observed concentrations  *- data reported earlier by Nawrot et al. (2020b) | | | | | | | | |

Nawrot, N, Wojciechowska, E., Matej-Łukowicz, K., Walkusz-Miotk, J., Pazdro, K., 2020b. Spatial and vertical distribution analysis of heavy metals in urban retention tanks sediments : a case study of Strzyza Stream. Environ. Geochem. Health 8. https://doi.org/10.1007/s10653-019-00439-8

**Table Supplement 6** Toxicity equivalent factor (TEQ) for individual PAHs and the sum of Σ16PAHs in sediment samples collected from RTS of the Oliwski and Strzyza streams in Gdansk, Poland

| **Sampling point** | **TEQ in mg/kg d.w.** | | | | | | | | | | | | | | | | **TEQ Σ16PAHs** |
| --- | --- | --- | --- | --- | --- | --- | --- | --- | --- | --- | --- | --- | --- | --- | --- | --- | --- |
|  | **NAPT** | **ACNY** | **ACEN** | **FLUR** | **PHEN** | **ANTH** | **FLTH** | **PYR** | **B(a)A** | **CHRY** | **B(b)F** | **B(k)F** | **B(a)P** | **INPY** | **D(ah)A** | **B(ghi)P** |  |
| **Oliwski Stream** |  |  |  |  |  |  |  |  |  |  |  |  |  |  |  |  |  |
| O1 IN | 9.20E-05 | 6.00E-05 | 2.29E-04 | 2.48E-04 | 2.76E-03 | 5.40E-03 | 3.60E-03 | 2.95E-03 | 1.53E-01 | 1.73E-03 | 1.34E-01 | 1.42E-02 | **1.37E+00** | 1.22E-01 | 2.57E-01 | 1.04E-02 | **2.08E+00** |
| O2 IN | 1.50E-04 | 7.60E-05 | 5.70E-05 | 1.25E-04 | 1.30E-03 | 3.20E-03 | 2.81E-03 | 2.09E-03 | 1.19E-01 | 1.57E-03 | 1.44E-01 | 1.28E-02 | **1.27E+00** | 1.58E-01 | 2.96E-01 | 1.32E-02 | **2.02E+00** |
| O2 OUT | 3.90E-05 | 1.70E-05 | 4.80E-05 | 5.50E-05 | 5.90E-04 | 1.15E-03 | 9.60E-04 | 7.60E-04 | 3.90E-02 | 4.90E-04 | 3.60E-02 | 3.40E-03 | 3.17E-01 | 2.95E-02 | 4.90E-02 | 2.84E-03 | 4.81E-01 |
| O3 IN | 2.00E-05 | 2.30E-05 | 2.40E-05 | 3.70E-05 | 4.00E-04 | 7.00E-04 | 7.10E-04 | 5.70E-04 | 2.80E-02 | 3.60E-04 | 2.24E-02 | 2.79E-03 | 2.29E-01 | 1.71E-02 | 2.90E-02 | 1.92E-03 | 3.33E-01 |
| O4 IN | 4.00E-06 | 4.00E-06 | 9.00E-06 | 1.40E-05 | 1.43E-04 | 4.20E-04 | 2.77E-04 | 2.27E-04 | 1.19E-02 | 1.44E-04 | 7.60E-03 | 9.20E-04 | 7.20E-01 | 3.40E-03 | 7.00E-03 | 4.00E-04 | 7.52E-01 |
| O5 IN | 4.90E-05 | 1.70E-05 | 2.10E-05 | 3.80E-05 | 3.23E-04 | 5.80E-04 | 5.80E-04 | 4.80E-04 | 2.35E-02 | 3.40E-04 | 2.51E-02 | 2.66E-03 | 2.07E-01 | 2.29E-02 | 3.50E-02 | 2.48E-03 | 3.21E-01 |
| **Strzyża Stream** | | | | | | | | | | | | | | | | | |
| S1 IN | 1.00E-06 | 2.00E-06 | 5.00E-06 | 9.00E-06 | 8.20E-05 | 1.00E-04 | 1.12E-04 | 8.80E-05 | 3.80E-03 | 5.40E-05 | 2.30E-03 | 3.00E-04 | 2.20E-02 | 1.10E-03 | 2.00E-03 | 1.60E-04 | 3.21E-02 |
| S2 IN | 1.00E-06 | 6.00E-06 | 1.70E-05 | 1.90E-05 | 1.87E-04 | 3.50E-04 | 2.96E-04 | 2.45E-04 | 1.25E-02 | 1.65E-04 | 9.00E-03 | 1.12E-03 | 9.00E-02 | 6.20E-03 | 1.10E-02 | 5.40E-04 | 1.32E-01 |
| S2 OUT | 1.30E-05 | 2.10E-05 | 1.10E-05 | 1.80E-05 | 2.71E-04 | 4.70E-04 | 6.00E-04 | 4.40E-04 | 2.90E-02 | 4.10E-04 | 3.40E-02 | 3.60E-03 | 3.02E-01 | 3.50E-02 | 6.60E-02 | 2.97E-03 | 4.75E-01 |
| S3 IN | 6.30E-05 | 4.00E-05 | 3.00E-05 | 7.20E-05 | 4.80E-04 | 1.17E-03 | 1.41E-03 | 1.22E-03 | 5.40E-02 | 8.20E-04 | 7.50E-02 | 6.60E-03 | 5.70E-01 | 8.20E-02 | 1.68E-01 | 8.40E-03 | 9.69E-01 |
| S3 OUT | 4.80E-05 | 3.20E-05 | 2.40E-05 | 5.90E-05 | 4.50E-04 | 9.90E-04 | 1.18E-03 | 1.00E-03 | 4.50E-02 | 7.20E-04 | 6.20E-02 | 5.40E-03 | 4.80E-01 | 6.60E-02 | 1.30E-01 | 6.40E-03 | 7.99E-01 |
| S4 IN | 1.20E-05 | 9.00E-06 | 3.00E-05 | 3.60E-05 | 2.69E-04 | 4.90E-04 | 3.80E-04 | 3.01E-04 | 1.48E-02 | 1.84E-04 | 1.06E-02 | 1.22E-03 | 1.06E-01 | 6.70E-03 | 1.10E-02 | 7.90E-04 | 1.53E-01 |
| **Bold** TEQ/ΣTEQ value greater than 1 | | | | | | | | | | | | | | | | | |

**Table Supplement 7** RQ ratios for individual PAHs’ and ΣPAHs calculated in reference to a) NCs – RQ_(NCs)_ and b) MPCs – RQ_(MPCs)_

| a) | **RQ_(NCs)_** | **O1 IN** | **O2 IN** | **O2 OUT** | **O3 IN** | **O4 IN** | **O5 IN** | **S1 IN** | **S2 IN** | **S2 OUT** | **S3 IN** | **S3 OUT** | **S4 IN** |
| --- | --- | --- | --- | --- | --- | --- | --- | --- | --- | --- | --- | --- | --- |
|  | **Napt** | 65.7 | 107.1 | 27.9 | 14.3 | 2.9 | 35 | 0.7 | 0.7 | 0.7 | 45 | 34.3 | 8.6 |
|  | **Acny** | 50 | 63.3 | 14.2 | 19.2 | 3.3 | 14.2 | 1.7 | 5 | 17.5 | 33.3 | 26.7 | 7.5 |
|  | **Acen** | 190.8 | 47.5 | 40 | 20 | 7.5 | 17.5 | 4.2 | 14.2 | 9.2 | 25 | 20 | 25 |
|  | **Flur** | 206.7 | 104.2 | 45.8 | 30.8 | 11.7 | 31.7 | 7.5 | 15.8 | 15 | 60 | 49.2 | 30 |
|  | **Phen** | 541.2 | 254.9 | 115.7 | 78.4 | 28 | 63.3 | 16.1 | 36.7 | 53.1 | 94.1 | 88.2 | 52.7 |
|  | **Anth** | 450 | 266.7 | 95.8 | 58.3 | 35 | 48.3 | 8.3 | 29.2 | 39.2 | 97.5 | 82.5 | 40.8 |
|  | **Flth** | 138.5 | 108.1 | 36.9 | 27.3 | 10.7 | 22.3 | 4.3 | 11.4 | 23.1 | 54.2 | 45.4 | 14.6 |
|  | **Pyr** | 2458.3 | 1741.7 | 633.3 | 475 | 189.2 | 400 | 73.3 | 204.2 | 366.7 | 1016.7 | 833.3 | 250.8 |
|  | **B(a)A** | 425 | 330.6 | 108.3 | 77.8 | 33.1 | 65.3 | 10.6 | 34.7 | 80.6 | 150 | 125 | 41.1 |
|  | **Chry** | 16.2 | 14.7 | 4.6 | 3.4 | 1.3 | 3.2 | 0.5 | 1.5 | 3.8 | 7.7 | 6.7 | 1.7 |
|  | **B(b)F** | 372.2 | 400 | 100 | 62.2 | 21.1 | 69.7 | 6.4 | 25 | 94.4 | 208.3 | 172.2 | 29.4 |
|  | **B(k)F** | 59.2 | 53.3 | 14.2 | 11.6 | 3.8 | 11.1 | 1.3 | 4.7 | 15 | 27.5 | 22.5 | 5.1 |
|  | **B(a)P** | 50.7 | 47 | 11.7 | 8.5 | 26.7 | 7.7 | 0.8 | 3.3 | 11.2 | 21.1 | 17.8 | 3.9 |
|  | **Inpy** | 45.2 | 58.5 | 10.9 | 6.3 | 1.3 | 8.5 | 0.4 | 2.3 | 13 | 30.4 | 24.4 | 2.5 |
|  | **D(ah)A** | 4.4 | 5 | 0.8 | 0.5 | 0.1 | 0.6 | 0 | 0.2 | 1.1 | 2.8 | 2.2 | 0.2 |
|  | **B(ghi)P** | 13.9 | 17.6 | 3.8 | 2.6 | 0.5 | 3.3 | 0.2 | 0.7 | 4 | 11.2 | 8.5 | 1.1 |
|  | **ΣPAHs** | 5088 | 3620 | 1263 | 896 | 376 | 801 | 134 | 388 | 748 | 1885 | 1559 | 515 |

| b) | **RQ_(MPCs)_** | **O1 IN** | | | **O2 IN** | | **O2 OUT** | | **O3 IN** | | **O4 IN** | | **O5 IN** | | **S1 IN** | | **S2 IN** | | **S2 OUT** | | **S3 IN** | | **S3 OUT** | | **S4 IN** |
| --- | --- | --- | --- | --- | --- | --- | --- | --- | --- | --- | --- | --- | --- | --- | --- | --- | --- | --- | --- | --- | --- | --- | --- | --- | --- |
|  | **Napt** | 0.7 | | | 1.1 | | 0.3 | | 0.1 | | 0 | | 0.4 | | 0 | | 0 | | 0 | | 0.5 | | 0.3 | | 0.1 |
|  | **Acny** | 0.5 | | | 0.6 | | 0.1 | | 0.2 | | 0 | | 0.1 | | 0 | | 0.1 | | 0.2 | | 0.3 | | 0.3 | | 0.1 |
|  | **Acen** | 1.9 | | | 0.5 | | 0.4 | | 0.2 | | 0.1 | | 0.2 | | 0 | | 0.1 | | 0.1 | | 0.3 | | 0.2 | | 0.3 |
|  | **Flur** | 2.1 | | | 1 | | 0.5 | | 0.3 | | 0.1 | | 0.3 | | 0.1 | | 0.2 | | 0.2 | | 0.6 | | 0.5 | | 0.3 |
|  | **Phen** | 5.4 | | | 2.5 | | 1.2 | | 0.8 | | 0.3 | | 0.6 | | 0.2 | | 0.4 | | 0.5 | | 0.9 | | 0.9 | | 0.5 |
|  | **Anth** | 4.5 | | | 2.7 | | 1 | | 0.6 | | 0.4 | | 0.5 | | 0.1 | | 0.3 | | 0.4 | | 1 | | 0.8 | | 0.4 |
|  | **Flth** | 1.4 | | | 1.1 | | 0.4 | | 0.3 | | 0.1 | | 0.2 | | 0 | | 0.1 | | 0.2 | | 0.5 | | 0.5 | | 0.1 |
|  | **Pyr** | 24.6 | | | 17.4 | | 6.3 | | 4.8 | | 1.9 | | 4 | | 0.7 | | 2 | | 3.7 | | 10.2 | | 8.3 | | 2.5 |
|  | **B(a)A** | 4.3 | | | 3.3 | | 1.1 | | 0.8 | | 0.3 | | 0.7 | | 0.1 | | 0.3 | | 0.8 | | 1.5 | | 1.3 | | 0.4 |
|  | **Chry** | 0.2 | | | 0.1 | | 0 | | 0 | | 0 | | 0 | | 0 | | 0 | | 0 | | 0.1 | | 0.1 | | 0 |
|  | **B(b)F** | 3.7 | | | 4 | | 1 | | 0.6 | | 0.2 | | 0.7 | | 0.1 | | 0.3 | | 0.9 | | 2.1 | | 1.7 | | 0.3 |
|  | **B(k)F** | 0.6 | | | 0.5 | | 0.1 | | 0.1 | | 0 | | 0.1 | | 0 | | 0 | | 0.2 | | 0.3 | | 0.2 | | 0.1 |
|  | **B(a)P** | 0.5 | | | 0.5 | | 0.1 | | 0.1 | | 0.3 | | 0.1 | | 0 | | 0 | | 0.1 | | 0.2 | | 0.2 | | 0 |
|  | **Inpy** | 0.5 | | | 0.6 | | 0.1 | | 0.1 | | 0 | | 0.1 | | 0 | | 0 | | 0.1 | | 0.3 | | 0.2 | | 0 |
|  | **D(ah)A** | 0 | | | 0.1 | | 0 | | 0 | | 0 | | 0 | | 0 | | 0 | | 0 | | 0 | | 0 | | 0 |
|  | **B(ghi)P** | 0.1 | | | 0.2 | | 0 | | 0 | | 0 | | 0 | | 0 | | 0 | | 0 | | 0.1 | | 0.1 | | 0 |
|  | **ΣPAHs** | | 48 | 33 | | 11 | | 5 | | 2 | | 4 | | 0 | | 2 | | 4 | | 14 | | 11 | | 3 | |
